# Biology and Genomics of an Historic Therapeutic *Escherichia coli* Bacteriophage Collection

**DOI:** 10.3389/fmicb.2017.01652

**Published:** 2017-08-30

**Authors:** Abiyad Baig, Joan Colom, Paul Barrow, Catherine Schouler, Arshnee Moodley, Rob Lavigne, Robert Atterbury

**Affiliations:** ^1^School of Veterinary Medicine and Science, University of Nottingham Sutton Bonington Campus Sutton Bonington, United Kingdom; ^2^Infectiologie et Santé Publique, Institut National de la Recherche Agronomique, Université François Rabelais de Tours Nouzilly, France; ^3^Department of Veterinary and Animal Sciences, Faculty of Health and Medical Sciences, University of Copenhagen Copenhagen, Denmark; ^4^Laboratory of Gene Technology, Department of Biosystems, KU Leuven Leuven, Belgium

**Keywords:** *Escherichia coli*, bacteriophage, therapeutics, one-step growth curve, transmission electron microscopy, phylogeny, DNA-dependent RNA polymerase

## Abstract

We have performed microbiological and genomic characterization of an historic collection of nine bacteriophages, specifically infecting a K1 *E. coli* O18:K1:H7 ColV^+^ strain. These phages were isolated from sewage and tested for their efficacy *in vivo* for the treatment of systemic *E. coli* infection in a mouse infection model by Smith and Huggins (1982). The aim of the study was to identify common microbiological and genomic characteristics, which co-relate to the performance of these phages in *in vivo* study. These features will allow an informed selection of phages for use as therapeutic agents. Transmission electron microscopy showed that six of the nine phages were *Podoviridae* and the remaining three were *Siphoviridae*. The four best performing phages *in vivo* belonged to the *Podoviridae* family. *In vitro*, these phages exhibited very short latent and rise periods in our study. In agreement with their microbiological profiles, characterization by genome sequencing showed that all six podoviruses belong to the *Autographivirinae* subfamily. Of these, four were isolates of the same species (99% identity), whereas two had divergent genomes compared to other podoviruses. The *Siphoviridae* phages, which were moderate to poor performers *in vivo*, exhibited longer latent and rise periods *in vitro*. Two of the three siphoviruses were closely related to each other (99% identity), but all can be associated with the *Guernseyvirinae* subfamily. Genome sequence comparison of both types of phages showed that a gene encoding for DNA-dependent RNA polymerase was only present in phages with faster replication cycle, which may account for their better performance *in vivo*. These data define a combination of microbiological, genomic and *in vivo* characteristics which allow a more rational evaluation of the original *in vivo* data and pave the way for the selection of phages for future phage therapy trails.

## Introduction

The rapid increase in antimicrobial resistance, is a major cause of concern for both the animal and human health, and threatens to render many clinically important antibiotics ineffective (Drulis-Kawa et al., 2012). There is now an urgent need for new antimicrobials and alternative approaches to infection control. Bacteriophages (phages) are one such approach with evidence of antibacterial properties, recently highlighted in 2016 by a joint United Kingdom Department of Health and Wellcome Trust-funded expert panel review of alternatives to antibiotics, and reviewed earlier (Drulis-Kawa et al., 2012; Nobrega et al., 2015).

Phages were discovered in the early twentieth century by Twort (1915) and d’Hérelle (1917). Soon after their discovery d’Herelle and others performed several trials using phages to treat bacterial infections in humans and animals. Although some of these trials were successful, they suffered from a lack of proper controls and the use of phage whose activity and reliability were unknown. This, together with the poor understanding at the time of the mechanism of bacterial pathogenesis, the nature of the interaction between bacteria and phages and the start of the antibiotic era, discredited the idea of using phages as antimicrobials in the West. The clinical use of phage continued in Eastern Europe, although studies published often lacked appropriate control groups and sometimes used phage alongside other antimicrobials, making it difficult to evaluate how much phage contributed to the efficacy of the treatment (Barrow and Soothill, 1997; Summers, 1999, 2001).

Phage therapy continues in Russia and Eastern Europe to this day (Expert Round Table on Acceptance and Therapy Re-implementation of Bacteriophage, 2016). In the West, phage therapy was revived in the 1980s by Smith and Huggins in a series of carefully controlled experiments using systemic *Escherichia coli* and Enterotoxigenic *E. coli* (ETEC) infections in mice, calves, pigs, and sheep (Smith and Huggins, 1980, 1982, 1983; Smith et al., 1987a,b). These studies highlighted that appropriate selection of phages and their application in infections where phage activity is likely to be optimal, can lead to a successful outcome.

Smith and Huggins’s (1982) study reported the isolation of phages which attached specifically to the K1 capsular antigen of *E. coli*. The capsular polysaccharides (K antigens) are linked to virulence in *E. coli* (Jann and Jann, 1987), and strains possessing the K1 antigen exhibit increased invasiveness and are associated with septicaemia and meningitis (Smith and Huggins, 1982; Silver and Vimr, 1990). The K1-specific phages were used to treat and prevent *E. coli* septicaemia or meningitis in mice (Smith and Huggins, 1982). Some phage-resistant mutants were recovered following the experiments but these were largely K1 negative, and thus avirulent. There were differences in therapeutic efficacy between the nine phages tested. Of the nine, one, phage R, was more effective than any of the antibiotic treatments used, which included several doses of streptomycin. In the present study, we have performed detailed microbiological and genomic characterization of the nine phages originally isolated by Smith and Huggins. Our findings define common phenotypic and genomic characteristics which can be related to the differences in the *in vivo* performance of these phage. These features could be useful in the selection of phage from closely related phage groups which are most likely to be effective biological control agents.

## Materials and Methods

### Bacterial Isolate and Phage Collection

The host bacterium *E. coli* O18:K1:H7 ColV^+^, referred to as MW, and the collection of nine phages used in this study are those isolated by Smith and Huggins (1982). Briefly, MW is a prototrophic, non-haemolytic human clinical *E. coli* isolated from the brain of a baby suffering from meningitis and which produces experimental septicaemia in mice, chickens and colostrum-deprived calves (Smith and Huggins, 1980, 1982). The nine phages were isolated from samples of crude sewage and from pig markets by Smith and Huggins (1982). In compliance with the new taxonomic classification for phages (Lavigne et al., 2008), the original names of phages have been modified and listed under ‘Phage name’ (**Table 1**) with the ‘Phage ID’ referring to the original name of phages (Smith and Huggins, 1982).

**Table 1:** Biological and genomic characteristics of phages used in this study.

Phage name^∗^	Phage ID^∗∗^	Number of dead mice ^∗∗∗^ and efficiency ^#^	Adsorption time (min)	Latent period (min)	Duration of rise period (min)	Duration of growth cycle (min)	Mean burst size (PFU per infected cell ±*SD*)	Proposed taxonomic classification	Genome size (bp)	G+C content (%)	Number of coding sequences (CDSs)
vB_EcoP_R	R	0	8	<2	4	14	19 ± 4.5	*Autographivirinae*	45230	45	61
vB_EcoP_D	D	1	8	<2	6	16	31 ± 11	*Autographivirinae*	45219	45	59
vB_EcoP_B	B	2	6	<2	2	10	30 ± 4.9	*Autographivirinae*	44307	45	56
vB_EcoP_C	C	3	6	<2	5	13	48 ± 2.1	*Autographivirinae*	45259	45	59
vB_EcoP_F	F	4	4	<2	8	14	213 ± 27	*T7virus*	39465	49	49
vB_EcoS_G	G	4	8	10	24	42	24 ± 11.3	*Guernseyvirinae*	41624	51	68
vB_EcoS_L	L	4	8	35	10	53	3 ± 0.0	*Guernseyvirinae*	41039	51	67
vB_EcoP_K	K	5	4	6	8	20	30 ± 2.8	*Sp6virus*	38025	45	46
vB_EcoS_P	P	9	8	10	10	28	315 ± 32	*Guernseyvirinae*	41185	51	62

*^∗^Systematic nomenclature (Kropinski et al., 2009) is used here to avoid confusion with other phage isolates with the same name. (‘vB’ = Virus of Bacteria; ‘Eco’ = isolation species *E. coli* and ‘P’ or ‘S’ for podovirus and siphovirus, respectively). ^∗∗^Phages are arranged in the decreasing order of effectiveness in treatment of MW *E. coli* infection in mice (Smith and Huggins, 1982). ^∗∗∗^Number of mice deaths 8 h post intramuscular treatment of MW *E. coli* infection (Smith and Huggins, 1982). ^#^0–3 mice deaths: best performing phages, 4–5 mice deaths: moderate performing phages, >5 mice deaths: poor performing phages.*

### Growth Rate of MW *E. coli*

The mid-exponential phase of MW *E. coli* was determined by growing the bacteria in LB broth. For this, LB broth was inoculated with an overnight culture of MW *E. coli* to an OD_600nm_ of 0.01. The first sample was taken at the point of inoculation and the culture was incubated shaking at 100 rpm at 37°C. Samples were taken out at hourly intervals for 7 h and finally after 24 h to enumerate the bacteria (Miles et al., 1938). For this, each sample was serially diluted in Phosphate Buffered Saline (PBS, Sigma–Aldrich) and 0.1 mL of each dilution was spread onto LBA plates which were then incubated overnight at 37°C. After incubation, the bacterial colonies were counted and CFU/mL calculated.

### Propagation of Phages in MW *E. coli*

All phage were propagated using MW as the host *E. coli* strain. Phage lysates were produced either in Luria-Bertani (LB) broth or on LB agar (LBA) plates (Sigma–Aldrich). Broth lysates were prepared by inoculating LB with an overnight culture of MW to achieve an optical density of 0.01 (OD_600nm_). The culture was incubated at 37°C, shaking (100 rpm) for 2 h until the cells reached mid-exponential growth phase (10^8^ CFU/mL) before infecting with a phage suspension at a multiplicity of infection (MOI) of 0.1. The culture was further incubated with gentle agitation (100 rpm) overnight at 37°C. Phage lysates were centrifuged at 5,000 × *g* for 10 min to pellet the cellular debris. The supernatant containing the phage was filtered using a 0.22 μm filter (Millipore). The phage were enumerated by serially diluting the lysate in SM buffer (50 mM Tris-Cl (pH 7.5), 0.1M NaCl, 8 mM MgSO_4_⋅7H_2_O and 0.01% gelatin). A 0.1 mL volume of each dilution was added separately to 3 mL of molten LBA containing 0.5% agar and 0.1 mL of an overnight culture of MW. This was poured onto LBA plates containing 1% agar which were then incubated overnight at 37°C. All phage lysates were stored at 4°C until required.

### Polyethylene Glycol (PEG) Precipitation

A high titre phage suspension (10^10^–10^11^ PFU/mL) was produced by PEG precipitation according to the method of Yamamoto et al. (1970). Briefly, overnight phage lysates were produced in 2L Erlenmeyer flasks. The flasks were removed from the incubator and supplemented with 1 μg/mL each of DNase and RNase (Sigma–Aldrich) before incubating at room temperature for 30 min. To allow the phage particles to dissociate from cellular debris, NaCl was added to a final concentration of 1M and the lysates were incubated on ice for 1 h. The cellular debris was removed by centrifugation at 11,000 × *g* (Beckman Coulter Avanti J-E series centrifuge) for 10 min at 4°C. The phage particles were precipitated by incubating the lysates overnight at 4°C with PEG 8000 (Sigma–Aldrich) to a final concentration of 10% w/v. The precipitated phage were pelleted by centrifugation at 35,000 × *g* for 15 min at 4°C. The phage pellet was re-suspended by soaking in SM buffer for 1 h at room temperature. Phage were extracted from PEG and cell debris by adding an equal volume of chloroform to the lysates which were mixed by gentle inversion for 30 s and the aqueous phase containing phage particles (upper layer) was separated by centrifugation at 13,000 × *g* (Thermo Electron Corporation Heraeus Pico 17 centrifuge) for 20 min.

### Phage Purification by Ultracentrifugation

High titre phage preparations were further purified using a caesium chloride (CsCl) gradient as described by Sambrook and Russell (2001) and adapted by Hooton et al. (2011). Briefly, CsCl was mixed with the phage lysate to a final concentration of 0.75 g/ml. The mixture was centrifuged at 240,000 × *g* for 24 h (15°C) (Beckman TL 100 series centrifuge). The phage band was extracted by piercing through the ultracentrifuge tube with a sterile 21-gauge hypodermic needle (Medisave). To remove the residual CsCl, the sample was filtered using an Amicon^®^ Ultra -0.5 mL 30 K membrane filter (Millipore) as per the manufacturer instructions.

### Transmission Electron Microscopy (TEM)

The sample (3 μL) was applied to a hydrophilic (freshly glow-discharged) carbon-coated Pioloform film coated 300 square mesh copper EM grid (Agar Scientific Ltd.) and left for 2 min to adsorb. Excess sample was removed with a small piece of Whatman No. 1 filter paper, leaving a thin film of sample on the grid surface. The grid was rinsed twice immediately by adding 5 μl distilled de-ionized water and removing excess as described above. One drop of uranyl acetate (1% and 0.22 μm filtered) was applied to the grid and the excess removed immediately. The grid was then allowed to dry. Once dry, the grids were observed on a JEOL JEM-1400 TEM with an accelerating voltage of 100 kV. Digital images were recorded using a SIS Megaview III digital camera with iTEM software.

### Phage Adsorption

For each anti-K1 phage, a MW *E. coli* culture at mid-exponential phase was infected at an MOI of 0.1. The adsorption rate for each phage was determined by taking samples every 2 min for up to 12 min. Bacterial cells with adsorbed phage were removed by centrifugation at 13,000 × *g*. The supernatant was then filtered using 0.45 μm filter (Millipore) and unbound phage were enumerated on LBA overlay containing 0.1 mL of MW *E. coli* on LBA plates, incubated overnight at 37°C. The adsorption period was considered as the time taken to achieve a 90% reduction in free, unbound phage (i.e., 1 log_10_ PFU/mL reduction from the initial titre) (Weber-Dąbrowska et al., 2016).

### One-Step Growth Curve

A mid-exponential phase MW culture was infected with the respective phage at an MOI of 0.1. The phage particles were allowed to adsorb to the time of 90% adsorption as calculated in the *in vitro* adsorption assay. The lysate was then diluted to 10^4^ PFU/ml in LB broth (Hyman and Abedon, 2009). Samples of the infected culture were collected every 2 min for 20 min for the *Podoviridae* or every 5 min for up to 60 min for the *Siphoviridae*, and unbound phages were enumerated on LBA overlays as described above. The burst size was calculated as the difference in the plaque forming unit (PFU) between the end of rise period and the latent period (Bolger-Munro et al., 2013).

### Genomic DNA Extraction and Sequencing

DNA extraction of the ultracentrifuged phage preparation was performed using the Wizard^®^ DNA clean-up system (Promega). Briefly, the phage sample was mixed thoroughly with the DNA Clean-up resin to break open the capsids and release DNA. The DNA and resin mix was filtered through a mini-column using a syringe barrel. The mini-column was washed with 80% isopropanol and the purified DNA was eluted with pre-warmed deionized water.

Genome sequencing of the phage was completed at NU-Omics facility, University of Northumbria. A DNA sequencing library of the phage genomic DNA (50 ng) was created using Illumina Nextera XT DNA preparation kit and libraries were sequenced using Illumina V2 2 bp × 250 bp chemistry on the Illumina MiSeq Platform.

### Phage Genome Sequence Analysis

Raw sequence reads in FastQ files were quality-filtered using Cutadapt (Martin, 2011) and Sickle (Joshi and Fass, 2011). Due to massive over sequencing a subset of reads was taken for each phage, to enable manageable assemblies with read depths of approximately 200× (quality filtered FastQ data was randomly subsampled using a Python script^1^). The sequence reads were *de novo* assembled using SPAdes (v3.6.2) (Bankevich et al., 2012) and in each case the phage genomes were resolved as a single contig. To circularize the phage genome, the beginning and the end of each assembled genome was joined with a short string of ‘Ns.’ The raw sequence reads from the quality filtered FastQ files were re-mapped to the above genome sequence, using Stampy (Lunter and Goodson, 2011) and visualized using Samtools and BCFtools (Li et al., 2009). Any missing nucleotide bases replaced the N’s, confirming the circularity of the phage genome. The intactness of each phage genome was also confirmed using PHAST (Zhou et al., 2011). Phage genomes were annotated using Prokka (Seemann, 2014), which uses Prodigal to predict coding sequences (CDSs) and also defines the ribosomal and transfer RNA genes (rRNA and tRNA), non-coding RNA and leader signal peptides. The nucleotide sequence of CDSs was used to perform blast searches against the Conserved Domain Database (CDD) (v3.15) of NCBI (Marchler-Bauer et al., 2015). The predicted functionality of the phage genomes was further improved by comparing the functional annotation generated by Prokka, CDD and the best matching published genome in NCBI. Nucleotide and protein comparisons were performed using the command line BLAST+ algorithm (Camacho et al., 2009). The nucleotide and amino acid sequence alignments were produced by ClustalW (CLUSTAL 2.1) (Thompson et al., 1994). The maximum likelihood phylogenetic analysis was performed using the generalized time-reversible (GTR) model to infer nucleotide evolution and Jones-Taylor-Thornton 1992 (JTT +CAT) model for protein evolution, with FastTree (Price et al., 2009). The nucleotide BLAST comparison figures were generated using Easyfig (Sullivan et al., 2011). The color coding for functional annotation was added using Inscape 0.92^2^. The sequence read data of phage genomes has been submitted to the European Nucleotide Archive (ENA) and the annotated phage genome sequences have been deposited in the NCBI GenBank database (Supplementary Table S1).

## Results

### Phage Morphology

**FIGURE 1:**
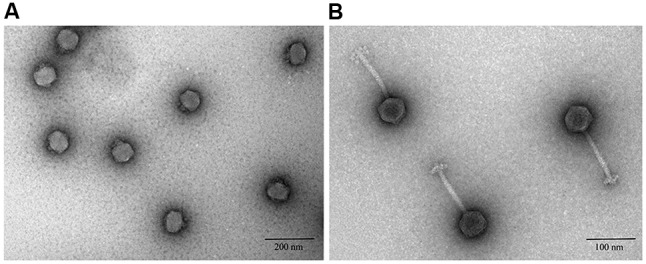
Negative staining transmission electron microscopic images of phages after ultracentrifugation. **(A)** *Podoviridae* phage R. **(B)** *Siphoviridae* phage P.

Transmission electron microscopy showed that six of nine phage studies used by Smith and Huggins (1982) belonged to the *Podoviridae* family (**Table 1**), which are known to have short, non-contractile tails (Ackermann, 2000). Phage vB_EcoP_R (R), the best performing phage reported by Smith and Huggins (**Figure 1A**), was also a podovirus with an icosahedral head (average diameter 46 ± 5 nm) and a very short tail (mean diameter 10 ± 5 nm). The remaining three phages belonged to the *Siphoviridae* family (**Table 1**). Phages from this family have a long flexible non-contractile tail with tail fibers (Fokine and Rossmann, 2014). For example, phage vB_EcoS_P (P) (**Figure 1B**), the poor performing phage in the *in vivo* study (Smith and Huggins, 1982) was a *Siphoviridae* phage of 150 ± 5 nm in size, with icosahedral head (average diameter 49 ± 5 nm) and long tail (average diameter 101 ± 5 nm).

### Growth Characteristics

Interestingly, high titre phage lysates (10^9^–10^10^ PFU/ml) for the *Siphoviridae* phages could only be produced using the plate lysate approach, whereas high titre lysates were easily achieved for the six *Podoviridae* phages from liquid lysates in LB broth. The time taken for the phages to adsorb to MW *E. coli* varied between 4 and 8 min. The best performing phages from the Smith and Huggins’s *in vivo* study, *Podoviridae* phages R, vB_EcoP_D (D), vB_EcoP_B (B) and vB_EcoP_C (C), exhibited the shortest latent period (<2 min), a rapid rise period (2–6 min) with a small burst size (19–48 PFU per infected cell). The growth cycle for these four podoviruses was between 10 and 16 min (**Table 1**). The *Podoviridae* phages vB_EcoP_F (F) and vB_EcoP_K (K) were moderate performers in the *in vivo* study (Smith and Huggins, 1982 and **Table 1**). Although, phage F had a latent period of <2 min like the best performing phages *in vivo* (R, D, B and C), it showed a large burst size (213 PFU per infected cell). Finally, phage K had the longest latent period of 8 min followed by a rise period of 8 min and the longest growth cycle of 20 min, compared to the other podoviruses. The three *Siphoviridae* phages [vB_EcoS_G (G), vB_EcoS_L (L), P] were moderate to poor performers in the *in vivo* experiment. These phages showed a longer latent (10–35 min) and rise period (10–24 min) and there was wider variation in the burst sizes between these phages, ranging between 3 and 315 PFU per infected cell. The duration of growth cycle in the three *Siphoviridae* phages was longer (28–53 min), compared with the *Podoviridae* phages (**Table 1**).

### Genome Sequence Analysis

Illumina MiSeq sequencing generated approximately 150–650 million basepairs (bp) per phage genome. A subsample of this data was used to assemble each phage genome at a read depth of approximately 200× (Supplementary Table S1).

Genome sequence analysis showed that the six *Podoviridae* phages had genome sizes that ranged between 38025 and 45259 bps, with a G+C content ranging between 45 and 49% and contained 46–61 CDSs. The remaining three *Siphoviridae* phages had genomes of 41039–41624 bps in size, with a higher G+C content of 51% and their genomes were composed of 62–68 CDSs (**Table 1**).

**FIGURE 2:**
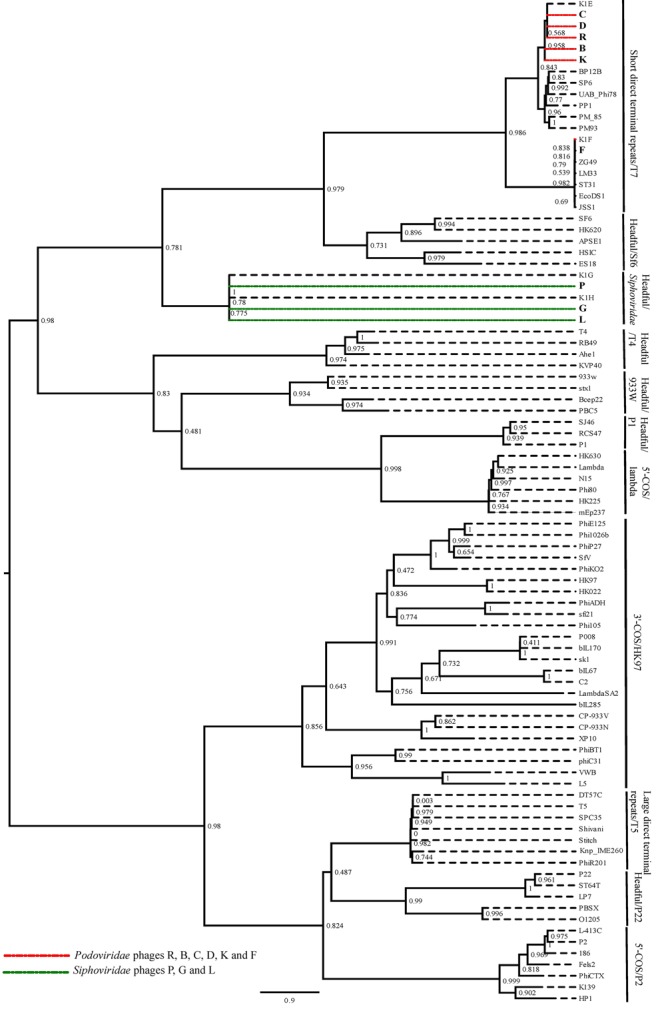
The maximum likelihood phylogenetic comparison of the amino acid sequence of the large terminase subunit of nine phages (Smith and Huggins, 1982) with the large terminase subunit from selected published tailed phages with well characterized DNA packaging strategy.

To predict the type of termini of nine phages, the amino acid sequence of the large terminase protein from these phages was compared with the terminase protein from published phages with well-studied DNA packaging mechanisms (Casjens and Gilcrease, 2009). The amino acid sequence of the large terminase subunit from *Escherichia* virus K1E, K1F, K1G and K1H, was also includes as the best matching published sequence to the *Podoviridae* and *Siphoviridae* phages in this study. The phylogenetic analysis showed that the six *Podoviridae* phages R, B, C, D, K and F were placed in the clade with the phages with short direct terminal repeats (**Figure 2**). The phages R, B, C, D and K were closely related to the *Escherichia* virus K1E and the phage F grouped with the *Escherichia* virus K1F. The *Siphoviridae* phages along with the *Escherichia* virus K1G and K1H formed a clade, and were closely related with the phages with different headful packaging strategies (**Figure 2**). Further comparison of the *Podoviridae* phages R, B, C, D and K with the *Escherichia* virus K1E showed that these phages have short direct terminal repeats of 250–289 bp in length with 92–96% sequence identity with the short direct terminal repeats in the *Escherichia* virus K1E. In the phage F, each short direct terminal repeat was 165 bp in length and showed 88% identity to the short direct terminal repeats in *Escherichia* virus K1F.

**FIGURE 3:**
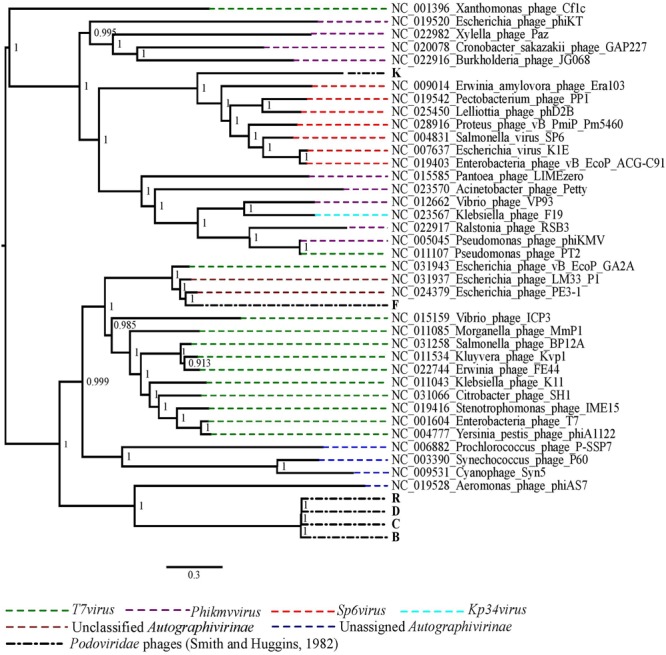
The maximum likelihood phylogenetic comparison of the *Podoviridae* phages (Smith and Huggins, 1982) to the selected published genomes of phage species from all genera of subfamily *Autographivirinae*.

The phylogenetic analysis comparing the *Podoviridae* phages with selected representative phage species from all genera of the subfamily *Autographivirinae* showed that the best performing phages *in vivo*, R, D, C and B grouped together and were closely related to *Aeromonas* phage phiAS7, an unassigned phage in the subfamily *Autographivirinae* (**Figure 3**). Phage K was more closely related to the phage species in the genus *Sp6virus* of the subfamily *Autographivirinae*. The *Escherichia* virus K1E was also included in the genus *Sp6virus*. Finally, phage F was included in a clade with two unclassified phages of subfamily *Autographivirinae* of which the best matched phage genome was of *Escherichia* phage PE3-1, a member of phage representing the *T7virus genus* (**Figure 3**).

**FIGURE 4:**
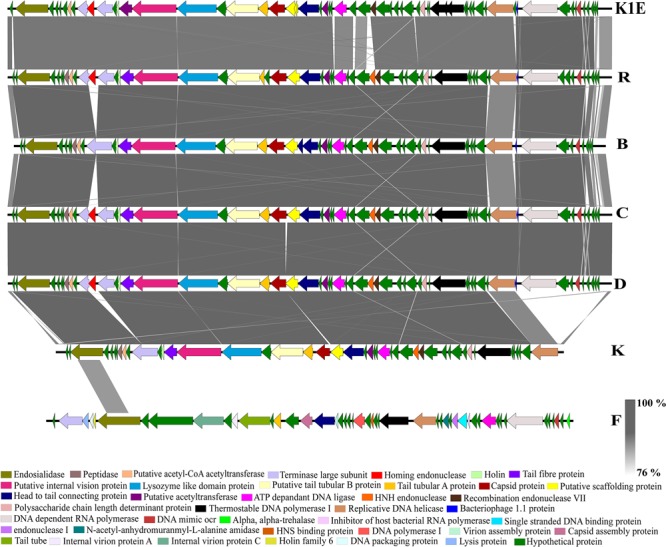
A BLASTn comparison of the *Escherichia* virus K1E and six *Podoviridae* phages, constructed with EasyFig.

The BLASTn comparison of the *Podoviridae* phage genomes using phage R as the reference showed an average of 99% identity with phages B, C and D (**Figure 4**). The moderately performing *Podoviridae* phages, K and F had divergent genomic content compared to the best performing *Podoviridae* phages R, D, C and B. Fourteen genes present in the best performing phage R were divergent from the genome of phage K. These included 10 genes with predicted hypothetical function and the remaining four genes encoding for DNA-dependent RNA polymerase, bacteriophage 1.1 protein, DNA mimic Ocr and a homing endonuclease. Finally, the genomic content of phage F differed most among the other six *Podoviridae* phages (**Figure 4** and Supplementary Table S2). Phylogeny and the nucleotide sequence comparison of the gene encoding DNA-dependent RNA polymerase from the *Podoviridae* phages R, B, C, D and F showed that the phages R, B, C and D were highly identical to each other (99–100%). Phage F only shared 47% nucleotide sequence identity with the phages, R, B, C and D based on the nucleotide sequence of DNA-dependent RNA polymerase (Supplementary Figures S1, S2).

**FIGURE 5:**
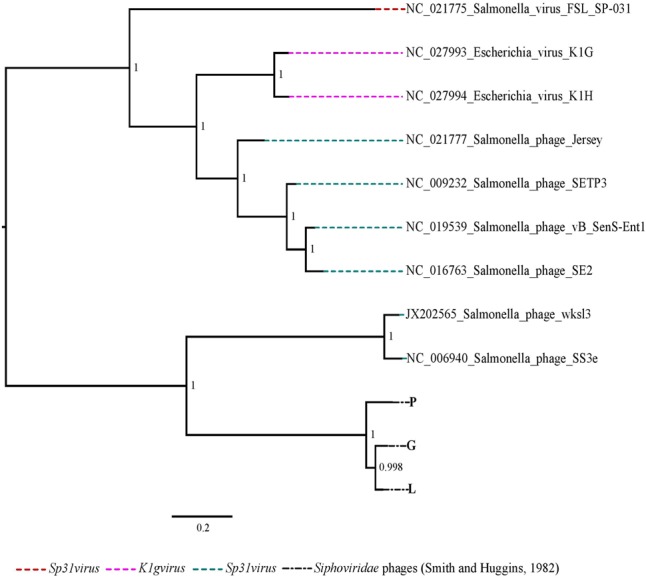
The maximum likelihood phylogenetic comparison of the *Siphoviridae* phages (Smith and Huggins, 1982) to the published genomes of phage species from all genera of subfamily *Guernseyvirinae*.

**FIGURE 6:**
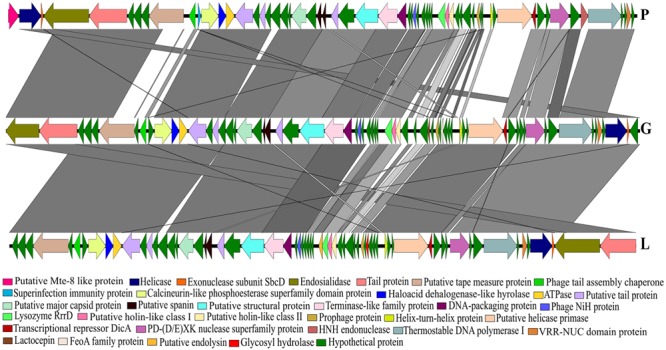
A BLASTn comparison of three *Siphoviridae* phages, constructed with EasyFig.

The phylogenetic analysis comparing the three *Siphoviridae* phages (P, G and L) with the published genomes from all genera of the subfamily *Guernseyvirinae* grouped the three phages in one clade, closely related to the two *Salmonella* phages (*Salmonella* phage wksl3 and *Salmonella* phage SS3e) in genus *Jerseyvirus* (**Figure 5**). Comparative analysis of the genome of the poor performing *Siphoviridae* phage P with that of the phages G and L showed that phage P contained nine genes which were divergent from phages G and L. Five of the nine genes had functions related to putative Mte8-like protein, phage tail assembly chaperone, superinfection immunity protein, putative helicase-primase, HNH endonuclease and the remaining four were predicted hypothetical function genes (**Figure 6** and Supplementary Table S3).

The gene encoding an endosialidase was present in all nine phages (**Figures 4, 6**). The phylogenetic analysis and comparison of nine phages based on the nucleotide sequence of the endosialidase gene showed that the *Podoviridae* phages R, B, C, D, K and F were closely related to each other with percentage sequence identity ranging between 92 and 100%. The *Siphoviridae* phages P, G and L shared >95% endosialidase nucleotide sequence identity with each other and were only distantly related to six *Podoviridae* phages with the nucleotide sequence identity of 52% (Supplementary Figures S3, S4).

## Discussion

Smith and Huggins (1982) tested the performance of nine K1 specific *E. coli* phages for the treatment of septicaemia, using mice model of infection. In these pioneering phage therapy experiments, the mice were infected by intramuscular or intracerebral route with MW *E. coli*. The K1 specific phages were administered intramuscularly to treat the infection. Interestingly, not all phages tested were equally effective and showed a gradient of treatment efficiency, noted after 8 h interval between infection and treatment (Smith and Huggins, 1982 and **Table 1**). From the best performing phages, phage R was investigated further and was more effective in treatment than multiple doses of either tetracycline, trimethoprim, ampicillin or chloramphenicol. It was also reported that phage R and the other of the more effective phages cleared broth cultures of the K1 positive strain more quickly than those which were less effective (Smith and Huggins, 1982).

In the present study, we report the microbiological and genomic characteristics of these nine K1 specific *E. coli* phages and relate these characteristics to their performance in the *in vivo* study. It was interesting to observe that even though all nine phages were K1-specific, morphologically they belonged to two different phage families (*Podoviridae* and *Siphoviridae*) (**Figure 1**). There were also differences between the podoviruses and siphoviruses based on their growth profiles. The best performing phages in Smith and Huggins’s *in vivo* study belonged to the *Podoviridae* family, with a faster replication cycle characterized by shorter latent and rise periods. In contrast, the *Siphoviridae* phages which were moderate to poor performers, exhibited longer latent and rise periods (**Table 1**). These differences were observed in the bacterial growth media (LB) unlike the report by Bull et al. (2002) in which the differences in the replication of K1-specific and non-K1 phage could only be observed in mouse sera. This suggests that phenotypic differences between these phages can be observed without the need for any specialist growth media. In a study of T7-like phages, a phage KPO_1_K_2_, capable of infecting *Klebsiella pneumoniae* isolate B5055, was a *Podoviridae* phage and was proposed as a potential therapeutic agent against this pathogen. High titres of phage KPO_1_K_2_ were observed in the kidney and urinary bladder 6 h after intraperitoneal injection in infected mice. The growth profile of PhiKPO_1_K_2_ showed a latent period of 15 min and a burst size of 140 (Verma et al., 2009). Another study describing the characterization of lytic phages for the treatment of multidrug resistant *Klebsiella pneumoniae* showed that both *Podoviridae* and *Siphoviridae* phages had a similar latent period of 15 min and a burst size of ∼50–60 (Kęsik-Szeloch et al., 2013). Although these studies suggest a longer latent period for being the best phage, our study suggests that the *Autographivirinae* phages that replicate faster in liquid culture, performed better in the mouse infection model. In contrast, the *Guernseyvirinae* phages that replicated more slowly due to longer time (latent period) required to assemble into complete phage particles performed poorly *in vivo*. This is also highlighted by the fact that it was difficult to produce high titre preparations of the *Siphoviridae* phages in the liquid LB media, requiring culture on LBA plates. It remains to be seen whether this is a general phenomenon for other phages or it is just restricted to the phages of these taxonomic groups.

A characteristic of K1 phage genomes is the presence of a gene encoding for endosialidase which accounts for the phage specificity to the K1 capsular antigen of the K1 *E. coli* strains (Tomlinson and Taylor, 1985; Mushtaq et al., 2005). The predicted gene encoding an endosialidase was present in all nine K1 phages confirming the specificity of these phages to the K1 *E. coli* strain. The phylogenetic placement of the four best performing phages (R, D, B and C) and the moderate performing phage F as closely related to the unassigned and unclassified members of the subfamily *Autographivirinae* suggests that these phages have divergent genomes compared to the phages in other genera within this subfamily. However, phage K which was a moderate performing phage of all *Podoviridae* phages had a more resolved position in the phylogeny as being closely related to the phages from genus *Sp6virus* which includes the *Escherichia* virus K1E. The three *Siphoviridae* phages were more closely related to the *Salmonella* phages in the genus *Jerseyvirus* which reflects that these phages may also infect *Salmonella* strains.

The best performance of *Podoviridae* phages R, D, B, and C *in vivo* could be associated with a combination of their rapid replication cycle and the presence of the gene encoding for DNA-dependent RNA polymerase in the genome of these phages. Although the phage K was phylogenetically closely related to the *Escherichia* virus K1E, many genes in the genome of this phage were divergent from the *Escherichia* virus K1E, including the gene encoding for DNA-dependent RNA polymerase suggesting that phage K had diverged from the phages in the genus *Sp6virus*. In phage T7, the phage-dependent RNA polymerase and the bacteriophage 1.1 protein are the early gene products which play a role in regulating host transcription and protein synthesis (McAllister and Barrett, 1977). The phage T7 RNA polymerase is also directly involved in phage DNA replication (Hinkle, 1980). If the gene encoding phage-dependent RNA polymerase is functional, it enables a rapid shut-off of host transcription by 5 min after infection, thus allowing a much faster replication cycle of the phages. In the absence of this gene, shut-off of host transcription is delayed and is instead activated by the bacteriophage 1.1 protein after 15 min of infection. In the absence of all early gene products, the host shut-off is observed at least 30 min after the infection (McAllister and Barrett, 1977). The moderate and poor performing phages in this study lacked these genes. The absence of these genes may be responsible for the longer latent period observed and thus the poor performance of these phages *in vivo*, as more time would be required to shut-off the host transcription. The moderate performance of phage F *in vivo* may also be linked to its different genomic content compared to the four best performing phages (R, D, B and C) and a large burst size. Similarly, the presence of the gene encoding the superinfection immunity protein in the *Siphoviridae* phage P, which is a characteristic of prophages (Brüssow and Kutter, 2005) may also explain its poor performance *in vivo*. Selecting phage based on the phenotypic and genomic characteristics may not guarantee success *in vivo* (Tsonos et al., 2014) but if common phenotypic and genomic features can be identified then these can certainly be used to make targeted phage selection and could also help in reducing the number of animals used in experiments. In the K1-specific phages, the presence of the gene encoding for DNA-dependent RNA polymerase only in the genomes of phages with very fast replication cycle and their best performance *in vivo* suggests the combination of these phenotypic and genomic characteristics might be used as a marker for selection of suitable phages for phage therapy trails in the future.

## Author Contributions

RA, PB, CS, AM, and RL conceived the study. AB, JC, PB, and RA produced the data. AB and JC analyzed the data. AB, JC, PB, CS, AM, RL, and RA wrote the paper.

## Conflict of Interest Statement

The authors declare that the research was conducted in the absence of any commercial or financial relationships that could be construed as a potential conflict of interest.

## Footnotes

1 https://github.com/fjruizruano/ngs-protocols/blob/master/fastq-pe-random.py

2 https://inkscape.org/en/
