# Supplementary material for: Biology and Genomics of an Historic Therapeutic Escherichia coli Bacteriophage Collection
Source: Front Microbiol. 2017 Aug 30;8:1652. doi: 10.3389/fmicb.2017.01652 (PMC5582158; doi:10.3389/fmicb.2017.01652)
Supplement: Supplementary file 2 [file Image_2.PDF]

```
F_podovirus     1 ATGAGCGTCATCTCTATTGACAAACACGACTTCTCTGATGTGTCGAACGCCATTGAGCCG
B_podovirus     1 ------------------------------------------------------------
R_podovirus     1 ------------------------------------------------------------
D_podovirus     1 ------------------------------------------------------------
C_podovirus     1 ------------------------------------------------------------
consensus       1

F_podovirus    61 TTTAACCTGCTGGCCGACCACTACGGGCAAGACCTTGCAGTCAAACAGCTTCAGCTTGAG
B_podovirus     1 ------------------------ATGCAAGACCTACACGCTATTCAACTTCAACTTGAA
R_podovirus     1 ------------------------ATGCAAGACCTACACGCTATTCAACTTCAACTTGAA
D_podovirus     1 ------------------------ATGCAAGACCTACACGCTATTCAACTTCAACTTGAA
C_podovirus     1 ------------------------ATGCAAGACCTACACGCTATTCAACTTCAACTTGAA
consensus      61                         ..*********....*..*..**.*****.*****.

F_podovirus   121 CATGAGGCATACACTGAAGGCGAGCGACGTTTCATCAAAAACCTTGAGCGACAGACTGAG
B_podovirus    37 GAAGAAATGTTTAACGGCGGTATCCGTCGCTTTGAAGCGGACCAACAACGCCAGATTGCA
R_podovirus    37 GAAGAAATGTTTAACGGCGGTATCCGTCGCTTTGAAGCGGACCAACAACGCCAGATTGCA
D_podovirus    37 GAAGAAATGTTTAACGGCGGTATCCGTCGCTTTGAAGCGGACCAACAACGCCAGATTGCA
C_podovirus    37 GAAGAAATGTTTAACGGCGGTATCCGTCGCTTTGAAGCGGACCAACAACGCCAGATTGCA
consensus     121 .*.**....*..*..*..**....**.**.**.......***...*.**.****.**..

F_podovirus   181 CGCGGGGAACTGGCAGACAATCAGGTCGCCAAGCCTTTGATGCAGACTCTGGTCCCTAAG
B_podovirus    97 TCCGGTAATGAATCAGACACGGCATGGAATCGCCGCTTATTGTCCGAACTAATCGCACCT
R_podovirus    97 TCCGGTAATGAATCAGACACGGCATGGAATCGACGCTTATTGTCCGAACTAATCGCACCT
D_podovirus    97 TCCGGTAATGAATCAGACACGGCATGGAATCGACGCTTATTGTCCGAACTAATCGCACCT
C_podovirus    97 TCCGGTAATGAATCAGACACGGCATGGAATCGACGCTTATTGTCCGAACTAATCACACCT
consensus     181 ..***..*.....******.............*..**..**......**..**.*....

F_podovirus   241 ATTGCGCAAGCCGTCAGGGAGTGGCATGAAGGTCCAGACGGGAAGCTGTCAACCTCTCGT
B_podovirus   157 ATGGCGGAAGGTATTCAGGCTTATAAGGAAGAGTACGAAGGTAAAAGAGGCCGCGCACCG
R_podovirus   157 ATGGCGGAAGGTATTCAGGCTTACAAGGAAGAATACGAAGGCAAGAGAGGTCGTGCACCA
D_podovirus   157 ATGGCGGAAGGTATTCAGGCTTACAAGGAAGAATACGAAGGCAAGAGAGGTCGTGCACCA
C_podovirus   157 ATGGCGGAAGGTATTCAGGCTTACAAGGAAGAATACGAAGGCAAGAGAGGTCGTGCACCA
consensus     241 **.***.***...*...**..*...*.****.....**.**.**..........*.*..

F_podovirus   301 CCTAGCGTAGCGTTCACC------------------------------------ATGTTA
B_podovirus   217 CGTGCATTAGCTTTCATTAACTGCGTAGAAAACGAAGTAGCAGCATATATCACCATGAAA
R_podovirus   217 CGTGCATTAGCTTTCATTAACTGCGTAGGAAACGAAGTGGCAGCATATATCACCATGAAA
D_podovirus   217 CGTGCATTAGCTTTCATTAACTGCGTAGGAAACGAAGTGGCAGCATATATCACCATGAAA
C_podovirus   217 CGTGCATTAGCTTTCATTAACTGCGTAGGAAACGAAGTGGCAGCATATATCACCATGAAA
consensus     301 *.*....****.****......................................***..*

F_podovirus   325 AGCACTGAAGAGAAGGCCGTCAAGGACCGCTCTCTG----CGTATCTCTTGCGAGTCTGC
B_podovirus   277 ATCGTTATGGACATGCTGAATAC-AGACGTTACCTTACAAGCCATCGCTATGAATGTAGC
R_podovirus   277 ATCGTTATGGATATGCTGAATAC-AGACGTTACCTTACAAGCTATCGCTATGAATGTAGC
D_podovirus   277 ATCGTTATGGATATGCTGAATAC-AGACGTTACCTTACAAGCTATCGCTATGAATGTAGC
C_podovirus   277 ATCGTTATGGATATGCTGAATAC-AGACGTTACCTTACAAGCTATCGCTATGAATGTAGC
consensus     361 *.*..*...**.*.*......*. ...**.*..**........***.**....*....**

F_podovirus   381 TGCGGTCATCATACTGAAGGTCATACTCTCCAAGCTGGTCAAGCCTGAAGGGATACCGAT
B_podovirus   336 GGACCGCATCGAAGACCAAGTACGTTTTAGCAAGCTGGAAGGTCACGCAGCTAAGTACTT
R_podovirus   336 AGACCGCATCGAGGACCAAGTACGTTTTAGCAAGCTGGAAGGTCACGCAGCTAAGTACTT
D_podovirus   336 AGACCGCATCGAGGACCAAGTACGTTTTAGCAAGCTGGAAGGTCACGCAGCTAAGTACTT
C_podovirus   336 AGACCGCATCGAGGACCAAGTACGTTTTAGCAAGCTGGAAGGTCACGCAGCTAAGTACTT
consensus     421 .*....****.......*.**.....*...*******.....*..*.**..*......*

F_podovirus   441 TACACCGATGGCCTCCGCGATAGGCCGCACACTTG-AGGACGAAATCCGCTTCGGTCGCA
B_podovirus   396 TGAGAA-----AGTGAAGAAGTCGCTTAAGGCTAGCAAGACTAAATCTTAT----CGTCA
R_podovirus   396 TGAGAA-----AGTGAAGAAGTCGCTTAAGGCTAGCAAGACTAAATCTTAT----CGTCA
D_podovirus   396 TGAGAA-----AGTGAAGAAGTCGCTTAAGGCTAGCAAGACTAAATCTTAT----CGTCA
C_podovirus   396 TGAGAA-----AGTGAAGAAGTCGCTTAAGGCTAGCAAGACTAAATCTTAT----CGTCA
consensus     481 *.....     ..*.....*...**...*..**.*.*.***.*****...*    ...**
```

```
F_podovirus  500 TCCGTGACAAAGAGAAAGAGCACTTCA---AGAAGGCGATAGCTGATAACCTGAATAAGC
B_podovirus  447 TGCGCACAACGTAGCGGTAGTAGCTGAGAAATCTGTTGCTGACCGTGACGCGGATTTC-T
R_podovirus  447 TGCGCACAACGTAGCGGTAGTAGCTGAGAAATCTGTTGCTGACCGTGACGCGGATTTC-T
D_podovirus  447 TGCGCACAACGTAGCGGTAGTAGCTGAGAAATCTGTTGCTGACCGTGACGCGGATTTC-T
C_podovirus  447 TGCGCACAACGTAGCGGTAGTAGCTGAGAAATCTGTTGCTGACCGTGACGCGGATTTC-T
consensus    541 *.**....*...**....**.*..*.*...*...*..*.*..*.*..*..*.**.*.. .

F_podovirus  557 GGGCCGGGGCGTCCTACAAGAAAGCCTACATGCAAGCGGTCGAGGCGTCCATGCTGGAGC
B_podovirus  506 CCCGTTGGGAGGCATGGCCTAAAGACACCTTGCTGCAAATCGGTATGACCTTGCTCGAAA
R_podovirus  506 CCCGTTGGGAGGCATGGCCTAAAGACACCTTGCTGCAAATCGGTATGACCTTGCTCGAAA
D_podovirus  506 CCCGTTGGGAGGCATGGCCTAAAGACACCTTGCTGCAAATCGGTATGACCTTGCTCGAAA
C_podovirus  506 CCCGTTGGGAGGCATGGCCTAAAGACACCTTGCTGCAAATCGGTATGACCTTGCTCGAAA
consensus    601 ......***.*.*.*.....****.*..*.***......***....*.**.****.**..

F_podovirus  617 AAGG----CCAACTGGAAGA-TGCGTGGGGGACTTGGAGTCCTACAGAGGCTGTCCACGT
B_podovirus  566 TATTGGAGAATAGTGTATTCTTCAATGGTCAACCTGTCTTCCTTCGTACCTTG-------
R_podovirus  566 TATTGGAGAATAGTGTATTCTTCAATGGTCAACCTGTTTTCCTTCGTACCTTG-------
D_podovirus  566 TATTGGAGAATAGTGTATTCTTCAATGGTCAACCTGTTTTCCTTCGTACCTTG-------
C_podovirus  566 TATTGGAGAATAGTGTATTCTTCAATGGTCAACCTGTTTTCCTTCGTACCTTG-------
consensus    661 .*.........*.**.*....*...***...**.**...****.*..*...**

F_podovirus  672 TGGCATCAAGATGCTTGAGATTGTCATCCAGTCAACACAACTGGTCGAGCTTAAGCGGTA
B_podovirus  619 ---------------------------------CGCACTAACGGCGGAAAGCATGGTGTT
R_podovirus  619 ---------------------------------CGCACTAACGGCGGAAAGCATGGTGTT
D_podovirus  619 ---------------------------------CGCACTAACGGCGGAAAGCATGGTGTT
C_podovirus  619 ---------------------------------CGCACTAACGGCGGAAAGCATGGTGTT
consensus    721                                  ..***.*..**..**....*.*..**.

F_podovirus  732 CGGTGCTGGCAATGCAGCGGCTGACGTAGAGATGGTCCACCTGTCGGACTTCTGGGTCAA
B_podovirus  646 TACTATTTACAGACCAGTGAACATGTTGGAGAGTGGATAAC---------TGCATTTAAG
R_podovirus  646 TACTATTTACAGACCAGTGAACATGTTGGCGAGTGGATAAC---------TGCATTTAAG
D_podovirus  646 TACTATTTACAGACCAGTGAACATGTTGGCGAGTGGATAAC---------TGCATTTAAG
C_podovirus  646 TACTATTTACAGACCAGTGAACATGTTGGCGAGTGGATAAC---------TGCATTTAAG
consensus    781 ...*..*..**...***.*.......*.*.**..*...*.*         *.....*.*.

F_podovirus  792 GAAGATGGCACAACGAGGATTCAGCCTTGCGGGTATCGCTCCAGTCTACCAGCCTTGCGT
B_podovirus  697 GAACATG-------------------TAGCGCAGCTCAGCCCTGCCTATGCACCTTGCGT
R_podovirus  697 GAACATG-------------------TAGCGCAGCTCAGCCCTGCCTATGCACCTTGCGT
D_podovirus  697 GAACATG-------------------TAGCGCAGCTCAGCCCTGCCTATGCACCTTGCGT
C_podovirus  697 GAACATG-------------------TAGCGCAGCTCAGCCCTGCCTATGCACCTTGCGT
consensus    841 ***.***                   *.***....**...**.*.***....*******

F_podovirus  852 CGTTCCACCTAAGCCGTGGACTGGTGTCGTAGGTGGTGGG--TACTGGGCCAAAGGTCGC
B_podovirus  738 TATACCTCCCCGCCCTTGGGTATCACCTTTTAACGGTGGGTTTCATACTGAGAAAGTAGC
R_podovirus  738 TATACCTCCCCGCCCTTGGGTATCACCTTTTAACGGTGGGTTTCATACTGAGAAAGTAGC
D_podovirus  738 TATACCTCCCCGCCCTTGGGTATCACCTTTTAACGGTGGGTTTCATACTGAGAAAGTAGC
C_podovirus  738 TATACCTCCCCGCCCTTGGGTATCACCTTTTAACGGTGGGTTTCATACTGAGAAAGTAGC
consensus    901 ..*.**.**....**.***..........*....******..*..*......**.**.**

F_podovirus  910 AGACCGTTACCATTGATTCGCTTAGGGTCTAAGTCTGCGG-TAGCACGTTATGAAGACGT
B_podovirus  798 AAGCCGTATTCGTCTGGTAA---AAGGCAATCGTGAGCACGTCCGCAAGCTGACCAAAAA
R_podovirus  798 AAGCCGTATTCGTCTGGTAA---AAGGCAATCGTGAGCACGTCCGCAAGCTGACCAAAAA
D_podovirus  798 AAGCCGTATTCGTCTGGTAA---AAGGCAATCGTGAGCACGTCCGCAAGCTGACCAAAAA
C_podovirus  798 AAGCCGTATTCGTCTGGTAA---AAGGCAATCGTGAGCACGTCCGCAAGCTGACCAAAAA
consensus    961 *..****...*.*....*..   *.**.....**..**...*..............*...

F_podovirus  969 GTACATGCCTGAAGTCTATGACGCTGTGAACATCATCCAAAATACACCTTGGAAAGTGAA
B_podovirus  855 GCAAATGCCAGCCGTTTATAAGGCTGTTAACGCTTTGCAGGCAACTAAGTGGCAGGTTAA
R_podovirus  855 GCAAATGCCAGCCGTTTATAAGGCTGTTAACGCTTTGCAGGCAACTAAGTGGCAGGTTAA
D_podovirus  855 GCAAATGCCAGCCGTTTATAAGGCTGTTAACGCTTTGCAGGCAACTAAGTGGCAGGTTAA
C_podovirus  855 GCAAATGCCAGCCGTTTATAAGGCTGTTAACGCTTTGCAGGCAACTAAGTGGCAGGTTAA
consensus   1021 *.*.*****.*..**.***.*.*****.***....*.**....**....***.*.**.**
```

```
F_podovirus 1029 CAAGGAGGTATTGGACGTGGTGAACATGGTCGAGAAGCTGAACAACACGCCTATTGATGA
B_podovirus  915 TAAAGAGGTTTTACAGGTTGTGGAGGACGTTATACGTCTAGA--CCTCGGCTATGGTGTA
R_podovirus  915 TAAAGAGGTTTTACAGGTTGTGGAGGACGTTATACGTCTAGA--CCTCGGCTATGGTGTA
D_podovirus  915 TAAAGAGGTTTTACAGGTTGTGGAGGACGTTATACGTCTAGA--CCTCGGCTATGGTGTA
C_podovirus  915 TAAAGAGGTTTTACAGGTTGTGGAGGACGTTATACGTCTAGA--CCTCGGCTATGGTGTA
consensus   1081 .**.*****.**..*.**.***.*....**.......**..*  .*.**.****.*...*

F_podovirus 1089 CATCCCTCAG-----------------------------------ATGGAACCGCTGAAG
B_podovirus  973 CCTTCCTTTAAACCACTCATCGACCGTGAGAACAAGCCAGCTAATCCGGTGCCGTTAGAG
R_podovirus  973 CCTTCCTTTAAACCACTCATCGACCGTGAGAACAAGCCAGCTAATCCGGTGCCGTTAGAG
D_podovirus  973 CCTTCCTTTAAACCACTCATCGACCGTGAGAACAAGCCAGCTAATCCGGTGCCGTTAGAG
C_podovirus  973 CCTTCCTTTAAACCACTCATCGACCGTGAGAACAAGCCAGCTAATCCGGTGCCGTTAGAG
consensus   1141 *.*.***...................................**..***.*..**

F_podovirus 1114 C-------------------------CTGAGGACTATGCTGGTGAGACCGA--GGAGGAA
B_podovirus 1033 TTCCAGCACCTGCGAGGACGTGAACTGAAAGAAATGTTAACACCGGAACAATGGCAAGCC
R_podovirus 1033 TTCCAGCACCTGCGAGGCCGTGAACTGAAAGAAATGTTAACACCGGAACAATGGCAAGCC
D_podovirus 1033 TTCCAGCACCTGCGAGGCCGTGAACTGAAAGAAATGTTAACACCGGAACAATGGCAAGCC
C_podovirus 1033 TTCCAGCACCTGCGAGGCCGTGAACTGAAAGAAATGTTAACACCGGAACAATGGCAAGCC
consensus   1201 ............................**.*.*.*........**.*.*..*.*.*..

F_podovirus 1147 CTCAAGGCATGGAAGAAGGCTGCT--GCTGGTATCTATCGCCGCGAGAAGGCCAGACAGT
B_podovirus 1093 TTCATCAACTGGAAAGGTGAATGCACCAAGCTGTATACCGCTGAGACTAAGCGCGGCAGC
R_podovirus 1093 TTCATCAACTGGAAAGGTGAATGCACCAAGCTGTATACCGCTGAGACTAAGCGCGGCAGC
D_podovirus 1093 TTCATCAACTGGAAAGGTGAATGCACCAAGCTGTATACCGCTGAGACTAAGCGCGGCAGC
C_podovirus 1093 TTCATCAACTGGAAAGGTGAATGCACCAAGCTGTATACCGCTGAGACTAAGCGCGGCAGC
consensus   1261 .***.....*****....*..........*.*.*.**.***.*.**..*.**..*.***.

F_podovirus 1205 CACGCAGATTATCACTGAGCTTTATCGTTAACCAAGCAAACAAGTTCTCTCAGTTCAAGG
B_podovirus 1153 AAATCGGCG--GCGACCGTCCGCATGGTAGGGCAGGCCCGTAAATACAGCCAATTTGACG
R_podovirus 1153 AAATCGGCG--GCGACCGTCCGCATGGTAGGGCAGGCCCGTAAATACAGCCAATTTGACG
D_podovirus 1153 AAATCGGCG--GCGACCGTCCGCATGGTAGGGCAGGCCCGTAAATACAGCCAATTTGACG
C_podovirus 1153 AAATCGGCG--GCGACCGTCCGCATGGTAGGGCAGGCCCGTAAATACAGCCAATTTGACG
consensus   1321 .*..*.*..  .*......*...**.**....**.**....**.*.*...**.**..*.*

F_podovirus 1265 CCATCTGGTTCCCGTACAACATGGATTGGCGAGGTCGTGTCTACGCTGTCCCGATGTT--
B_podovirus 1211 CAATATACTTCGTGTATGCTCTGGACAGCCGCAGCCGCGTCTACGCGCAATCTAGCACGC
R_podovirus 1211 CAATATACTTCGTGTATGCTCTGGACAGCCGCAGCCGCGTCTACGCGCAATCTAGCACGC
D_podovirus 1211 CAATATACTTCGTGTATGCTCTGGACAGCCGCAGCCGCGTCTACGCGCAATCTAGCACGC
C_podovirus 1211 CAATATACTTCGTGTATGCTCTGGACAGCCGCAGCCGCGTCTACGCGCAATCTAGCACGC
consensus   1381 *.**.*..***..***.....****..*.**..*.**.********.....*.*......

F_podovirus 1323 -CAACCCTCAGGGTAACGACATGCAGAAGGTCTCCTGACTCTGGCAGTCGGCAAGCC--
B_podovirus 1271 TCTCTCCGCAATCAAACGACTTAGGCAAGGCATTGCTCCGTTTTACCGAAGGGCAGCGTC
R_podovirus 1271 TCTCTCCGCAATCAAACGACTTAGGCAAGGCATTGCTCCGTTTTACCGAAGGGCAGCGTC
D_podovirus 1271 TCTCTCCGCAATCAAACGACTTAGGCAAGGCATTGCTCCGTTTTACCGAAGGGCAGCGTC
C_podovirus 1271 TCTCTCCGCAATCAAACGACTTAGGCAAGGCATTGCTCCGTTTTACCGAAGGGCAGCGTC
consensus   1441 .*...**.**....******.*....****...*.**...*.*..*.*..**..***...

F_podovirus 1380 -TATTGGTGCTGACGGTTTCAAATGGCTGAAGGTCCACGGTGCAAACTGTGCGGGTGTCG
B_podovirus 1331 TTGATAGCGCTGAGGCGCTTAAGTGGTTTTTGGTGAACGGGGCTAATAACTGGGGTTGGG
R_podovirus 1331 TTGATAGCGCTGAGGCGCTTAAGTGGTTTTTGGTGAACGGGGCTAATAACTGGGGTTGGG
D_podovirus 1331 TTGATAGCGCTGAGGCGCTTAAGTGGTTTTTGGTGAACGGGGCTAATAACTGGGGTTGGG
C_podovirus 1331 TTGATAGCGCTGAGGCGCTTAAGTGGTTTTTGGTGAACGGGGCTAATAACTGGGGTTGGG
consensus   1501 .*..*.*.*****.*...*.**.***.*...***..****.**.**......****...*

F_podovirus 1439 ATAAAGTCACCTTCGAGGAGCGCATCAAGTGGGTGGAAGACAACCA---CGAAAACATC-
B_podovirus 1391 ATAAGAAAACTTTTGACGTGCGCACCGCTAACGTGCTGGATGGTGAATTTCAAGACATGT
R_podovirus 1391 ATAAGAAAACTTTTGACGTGCGCACCGCTAACGTGCTGGATAGTGAATTTCAAGACATGT
D_podovirus 1391 ATAAGAAAACTTTTGACGTGCGCACCGCTAACGTGCTGGATAGTGAATTTCAAGACATGT
C_podovirus 1391 ATAAGAAAACTTTTGACGTGCGCACCGCTAACGTGCTGGATAGTGAATTTCAAGACATGT
consensus   1561 ****....**.**.**.*.*****.*......***...**.....*.....**.****..
```

```
F_podovirus 1495 --ATGGCTGCTGCTAAGGCACCGATGGATAGTATTGAGTGGTGGGCAAGTTAGACTCTC
B_podovirus 1451 GCCGCGACATTGCAGCGGATCCGCTGA---CCTTCACTCAATGGGTAAATGCCGACTCCC
R_podovirus 1451 GCCGCGACATTGCAGCGGATCCGCTGA---CCTTCACTCAATGGGTAAATGCCGACTCCC
D_podovirus 1451 GCCGCGACATTGCAGCGGATCCGCTGA---CCTTCACTCAATGGGTAAATGCCGACTCCC
C_podovirus 1451 GCCGCGACATTGCAGCGGATCCGCTGA---CCTTCACTCAATGGGTAAATGCCGACTCCC
consensus   1621 .....*....***...**..***.**.   ...*.......****..**....*****.*

F_podovirus 1553 CGTTCTGTTTCCTCGCGTTCTGCTTCGAGTATGCTGGCGTAATGC---------ACCACG
B_podovirus 1508 CTTACGGCTTCCTTGCATGGTGCTTTGAATATGCGCGTTATCTGGATGCACTGGATGAAG
R_podovirus 1508 CTTACGGCTTCCTTGCATGGTGCTTTGAATATGCGCGTTATCTGGATGCACTGGATGAAG
D_podovirus 1508 CTTACGGCTTCCTTGCATGGTGCTTTGAATATGCGCGTTATCTGGATGCACTGGATGAAG
C_podovirus 1508 CTTACGGCTTCCTTGCATGGTGCTTTGAATATGCGCGTTATCTGGATGCACTGGATGAAG
consensus   1681 *.*.*.*.*****.**.*..*****.**.*****..*.....**..........*..*.*

F_podovirus 1604 GCCTGTC------TTACTCCTGCTCGCTGCCGATAGCGTTCGATGGGTCCTGCTCTGGTA
B_podovirus 1568 GCACGCAAGACCAATTCATGACGCACCTCCCAGTCCATCAAGATGGTAGTTGTTCTGGTA
R_podovirus 1568 GCACGCAAGACCAATTCATGACGCACCTCCCAGTCCATCAAGATGGTAGTTGTTCTGGTA
D_podovirus 1568 GCACGCAAGACCAATTCATGACGCACCTCCCAGTCCATCAAGATGGTAGTTGTTCTGGTA
C_podovirus 1568 GCACGCAAGACCAATTCATGACGCACCTCCCAGTCCATCAAGATGGTAGTTGTTCTGGTA
consensus   1741 **..*.........*.*.........**.**..*.......*****....**.*******

F_podovirus 1658 TTCAGCACTTCAGTGCGATGCTTCGTGACCACATCGGTGGACATGCAGTAAACCTGACGC
B_podovirus 1628 TCCAGCACTACAGTGCTATGCTACGCGATGCAGTAGGTGCGAAAGCAGTAAACCTTAAGC
R_podovirus 1628 TCCAGCACTACAGTACTATGCTACGCGATGCAGTAGGTGCGAAAGCGGTAAACCTTAAGC
D_podovirus 1628 TCCAGCACTACAGTACTATGCTACGCGATGCAGTAGGTGCGAAAGCGGTAAACCTTAAGC
C_podovirus 1628 TCCAGCACTACAGTACTATGCTACGCGATGCAGTAGGTGCGAAAGCGGTAAACCTTAAGC
consensus   1801 *.*******.****.*.*****.**.**.....*.****...*.**.********.*.**

F_podovirus 1718 CATCCGGTAAGGTACAAGACATCTACCGCATTGTGTCTGACCGCATTGAGGAGGAGCTTA
B_podovirus 1688 CCTCTGACTCTCCTCAAGATATTTATGGTGCCGTTGCGCAGGTAGTAATTCAGAAGAATT
R_podovirus 1688 CCTCTGACTCCCCTCAAGATATTTATGGTGCCGTTGCGCAGGTAGTAATTCAGAAGAATT
D_podovirus 1688 CCTCTGACTCCCCTCAAGATATTTATGGTGCCGTTGCGCAGGTAGTAATTCAGAAGAATT
C_podovirus 1688 CCTCTGACTCCCCTCAAGATATTTATGGTGCCGTTGCGCAGGTAGTAATTCAGAAGAATT
consensus   1861 *.**.*........*****.**.**..*....**..*..*.....*.....**.**..*.

F_podovirus 1778 AAGTCCTGCTGATTAACGGTACTGACAACGAGATGGTCACTCACGAGGATAAGAAAACTG
B_podovirus 1748 ATGCATACATGAATGCAGAGGATGCGGAAACCTTCACTTCTGGCAGCGTGACTTTAACAG
R_podovirus 1748 ATGCATACATGAATGCAGAGGATGCGGAAACCTTCACTTCTGGCAGCGTGACTTTAACAG
D_podovirus 1748 ATGCATACATGAATGCAGAGGATGCGGAAACCTTCACTTCTGGCAGCGTGACTTTAACAG
C_podovirus 1748 ATGCATACATGAATGCAGAGGATGCGGAAACCTTCACTTCTGGCAGCGTGACTTTAACAG
consensus   1921 *.*......***.*...*....**...*.....*.....**..*...*..*....***.*

F_podovirus 1838 GTGAGATTACCGAACGTATCAAGCTGGGGACACGAGAGCTGGCCCGTCAGTGGCTGACAT
B_podovirus 1808 GTGCGG---------------AAC------TGCGTAGTATGGCTAGTGCGTGGGATATGA
R_podovirus 1808 GTGCAG---------------AAC------TTCGTAGCATGGCTAGTGCGTGGGATATGA
D_podovirus 1808 GTGCAG---------------AAC------TTCGTAGCATGGCTAGTGCGTGGGATATGA
C_podovirus 1808 GTGCAG---------------AAC------TTCGTAGCATGGCTAGTGCGTGGGATATGA
consensus   1981 ***...               *.*      ..**.....****..**..****...*...

F_podovirus 1898 ACGGTATGTCACGCAAGGTCACTAAGCGTTCAGTCATGACTCTGGCATACGGTCGAAAG
B_podovirus 1847 TAGGAATCACTCGCGGCCTGACCAAAAAGCCCGTAATGACACTACCTTATGGCAGCACAC
R_podovirus 1847 TAGGAATCACTCGCGGCCTGACCAAAAAGCCTGTAATGACACTACCTTATGGCAGCACAC
D_podovirus 1847 TAGGAATCACTCGCGGCCTGACCAAAAAGCCTGTAATGACACTACCTTATGGCAGCACAC
C_podovirus 1847 TAGGAATCACTCGCGGCCTGACCAAAAAGCCTGTAATGACACTACCTTATGGCAGCACAC
consensus   2041 ..**.**..*.***....*.**.**.....*.**.*****.**..*.**.**....*.*.

F_podovirus 1958 AGTACGGCTTCGCAGACCAAGTTTACGAGGACATCGTGATG-------------------
B_podovirus 1907 GTCTAACCTGCCGTGAGTCAGTGATTGATTATATCGTTGATTTAGAAGAAAAAGAGGCCC
R_podovirus 1907 GTCTAACCTGCCGTGAATCAGTGATTGATTATATCGTTGATTTGGAAGAAAAAGAGGCCC
D_podovirus 1907 GTCTAACCTGCCGTGAATCAGTGATTGATTATATCGTTGATTTGGAAGAAAAAGAGGCCC
C_podovirus 1907 GTCTAACCTGCCGTGAATCAGTGATTGATTATATCGTTGATTTGGAAGAAAAAGAGGCCC
consensus   2101 .......**.*...**...***....**..*.*****.......................
```

```
F_podovirus 1999 --CCAGCGATTGACTCAGGGTCTGGCGCTA--------------TGTTCACCG--------
B_podovirus 1967 AACGGGCTATTGCGGAAGGGCGTACCGCCAATCCTGTACACCCTTTTGATAATGACCGTA
R_podovirus 1967 AACGGGCTATTGCGGAAGGGCGTACCGCCAATCCTGTACATCCTTTTGATAATGACCGCA
D_podovirus 1967 AACGGGCTATTGCGGAAGGGCGTACCGCCAATCCTGTACATCCTTTTGATAATGACCGCA
C_podovirus 1967 AACGGGCTATTGCGGAAGGGCGTACCGCCAATCCTGTACATCCTTTTGATAATGACCGCA
consensus   2161 ..*..**.****....****..*..***.*.............*.**.*...........

F_podovirus 2036 ------------AGCCGAGCCAAGCGTCTCGCTTCATGGCTAAGATGATTTGGAAGCTG
B_podovirus 2027 AAGACAGCCTGACACCTAGCGCAGCTTATAACTATATGACAGCTTTAATCTGGCCTTCTA
R_podovirus 2027 AAGACAGCCTGACACCTAGCGCAGCTTATAACTATATGACAGCTTTAATCTGGCCTTCTA
D_podovirus 2027 AAGACAGCCTGACACCTAGCGCAGCTTATAACTATATGACAGCTTTAATCTGGCCTTCTA
C_podovirus 2027 AAGACAGCCTGACACCTAGCGCAGCTTATAACTATATGACAGCTTTAATCTGGCCTTCTA
consensus   2221 ..............**.***..***.*.*..**..***.*.....*.**.***....**.

F_podovirus 2084 TGAGCGTGACCGTAGTTGCTGCGGTTGACGCGATGAAGTGGCTTCAGGGTGCTGCCAAGC
B_podovirus 2087 TCTCGGAAGTGGTTAAAGCTCCTATAGTGGCAATGAAAATGATTCGTCAG--------CT
R_podovirus 2087 TTTCGGAAGTGGTTAAAGCTCCTATAGTGGCAATGAAAATGATTCGTCAG--------CT
D_podovirus 2087 TTTCGGAAGTGGTTAAAGCTCCTATAGTGGCAATGAAAATGATTCGTCAG--------CT
C_podovirus 2087 TTTCGGAAGTGGTTAAAGCTCCTATAGTGGCAATGAAAATGATTCGTCAG--------CT
consensus   2281 *....*.....**....***.*..*.*..**.*****...*.***.....        ..

F_podovirus 2144 TGCTGGCTGCTGAAGTGAAGGACAAGAAGACTGGAGAAATCCTGAAGCCTTGCCTTCCGG
B_podovirus 2139 TGCACGTTTCGCAGCTAAAAGAAACGAAGGCTTAGAATATCCCT----------------
R_podovirus 2139 TGCACGTTTCGCAGCTAAAAGAAACGAAGGCTTAGAATATCCCT----------------
D_podovirus 2139 TGCACGTTTCGCAGCTAAAAGAAACGAAGGCTTAGAATATCCCT----------------
C_podovirus 2139 TGCACGTTTCGCAGCTAAAAGAAACGAAGGCTTAGAATATCCCT----------------
consensus   2341 ***..*.*.*..*..*.**.**.*.****.**....*.****..

F_podovirus 2204 TACACTGGGTAACACCTGATGGTTTCCCGGTCTGGCA--GGAATACCGCAAGAAGGATAC
B_podovirus 2183 ------------TACCAACTGGCTTCATCTTGCAACAAAAGATAATGGCTACAGATATGC
R_podovirus 2183 ------------TACCAACTGGCTTCATCTTGCAACAAAAGATAATGGCTACCGATATGC
D_podovirus 2183 ------------TACCAACTGGCTTCATCTTGCAACAAAAGATAATGGCTACCGATATGC
C_podovirus 2183 ------------TACCAACTGGCTTCATCTTGCAACAAAAGATAATGGCTACCGATATGC
consensus   2401             .***...***.***....*....**...**..*..**.*.....**.*

F_podovirus 2262 CACTCGTCTGAATCTGATGTTCTTAGGGTCATTCAACCTTCAACCTACGGTCAACAAAGG
B_podovirus 2231 TCCGCGTATCCACTTG--CTTGATGGGTGAAATCAAGATGAGTCTCCAGATT--------
R_podovirus 2231 TCCGCGTATCCACTTG--CTTGATGGGTGAAATCAAGATGAGTCTCCAGATT--------
D_podovirus 2231 TCCGCGTATCCACTTG--CTTGATGGGTGAAATCAAGATGAGTCTCCAGATT--------
C_podovirus 2231 TCCGCGTATCCACTTG--CTTGATGGGTGAAATCAAGATGAGTCTCCAGATT--------
consensus   2461 ..*.***.*..*..**  .**..*.**...*.****..*....*....*.*.

F_podovirus 2322 CATGAAGAAAGAGCTGGACAAGCACAAGCAGGAGTCAGGCATTAGCCCGAACTTCGTCCA
B_podovirus 2281 GAAACAGATGTAGTTGAT-GAAACGGCAATGATGGGTGCGGCTGCTCCTAACTTCGTCCA
R_podovirus 2281 GAAACAGATGTAGTTGAT-GAAACGGCAATGATGGGTGCGGCTGCTCCTAACTTCGTCCA
D_podovirus 2281 GAAACAGATGTAGTTGAT-GAAACGGCAATGATGGGTGCGGCTGCTCCTAACTTCGTCCA
C_podovirus 2281 GAAACAGATGTAGTTGAT-GAAACGGCAATGATGGGTGCGGCTGCTCCTAACTTCGTCCA
consensus   2521 .*...***...**.**.. .*.........*..*...*....*...**.***********

F_podovirus 2382 CTCACAAGATGGTAGCCACCTACGCAAGACTGTAGTCCACACGCATCGCAAGTATGGCGT
B_podovirus 2340 TGGTCACGATGCTAGCCACCTGATTCTGACTGTATGTGACCTAGTG---GATAAAGGTAT
R_podovirus 2340 TGGTCACGATGCTAGCCACCTGATTCTGACTGTATGTGACCTAGTG---GATAAAGGTAT
D_podovirus 2340 TGGTCACGATGCTAGCCACCTGATTCTGACTGTATGTGACCTAGTG---GATAAAGGTAT
C_podovirus 2340 TGGTCACGATGCTAGCCACCTGATTCTGACTGTATGTGACCTAGTG---GATAAAGGTAT
consensus   2581 ....**.****.*********......*******....**......   .*..*.**..*

F_podovirus 2442 GATGTCATTCGCAGTGATTCATGATAGCTTCGGGACCATCCCGGCTGACGCTGAATATCT
B_podovirus 2397 TACATCCATTGCAGTCATCCATGATTCTTTCGGTACTCATGCAGGACGTACCGCAGACCT
R_podovirus 2397 TACATCCATTGCAGTCATCCATGATTCTTTCGGTACTCATGCAGGACGTACCGCAGACCT
D_podovirus 2397 TACATCCATTGCAGTCATCCATGATTCTTTCGGTACTCATGCAGGACGTACCGCAGACCT
C_podovirus 2397 TACATCCATTGCAGTCATCCATGATTCTTTCGGTACTCATGCAGGACGTACCGCAGACCT
consensus   2641 .*..**..*.*****.**.******...*****.**.....*.*......*.*.*.*.**
```

```
F_podovirus 2502 GTTCCGTGGTGTCCGTGAGACGATGGTCGAGACCTACCGCGACAACGATGTGCTGCTTGA
B_podovirus 2457 GCGGGATAGCTTAAGGGAAGAAATGGTTAAGATGTATCAAAACCATAATGCCCTGCAAAA
R_podovirus 2457 GCGGGATAGCTTAAGGGAAGAAATGGTTAAGATGTATCAAAACCATAATGCCCTGCAAAA
D_podovirus 2457 GCGGGATAGCTTAAGGGAAGAAATGGTTAAGATGTATCAAAACCATAATGCCCTGCAAAA
C_podovirus 2457 GCGGGATAGCTTAAGGGAAGAAATGGTTAAGATGTATCAAAACCATAATGCCCTGCAAAA
consensus   2701 *.....*.*..*..*.**....*****..***..**.*...**.*..***..****...*

F_podovirus 2562 CTTCTACGAGCAGTTTGAATACCAGCTTCACGAGAGCCAGCGCGATAAGTTGCCTGAGCT
B_podovirus 2517 CCTGCTAGATGTGCACGAAGAGCGTTGGTT---------AGTAGACACCGGAATCCAAGT
R_podovirus 2517 CCTGCTAGATGTGCACGAAGAGCGTTGGTT---------AGTAGACACCGGAATACAAGT
D_podovirus 2517 CCTGCTAGATGTGCACGAAGAGCGTTGGTT---------AGTAGACACCGGAATACAAGT
C_podovirus 2517 CCTGCTAGATGTGCACGAAGAGCGTTGGTT---------AGTAGACACCGGAATACAAGT
consensus   2761 *.*....**...*...***.*.*.......         ....**.*.........*..*

F_podovirus 2622 TCCGAAGAAAGGTAAACTGAATATCGAAGACATCTTGTCTTCAGACTTTGCATTCGCTTA
B_podovirus 2568 ACCAGAGCAAGGGGAGTTTGACCTTAACGAAATCTTAGTTTCAGACTATTGTTTCGCATA
R_podovirus 2568 ACCAGAGCAAGGGGAGTTTGACCTTAACGAAATCTTAGTTTCAGACTATTGCTTCGCATA
D_podovirus 2568 ACCAGAGCAAGGGGAGTTTGACCTTAACGAAATCTTAGTTTCAGACTATTGCTTCGCATA
C_podovirus 2568 ACCAGAGCAAGGGGAGTTTGACCTTAACGAAATCTTAGTTTCAGACTATTGCTTCGCATA
consensus   2821 .**..**.****..*..*..*..*..*.**.*****...********.*...*****.**

F_podovirus 2682 A

B_podovirus 2628 A
R_podovirus 2628 A
D_podovirus 2628 A
C_podovirus 2628 A
consensus   2881 *
```

**Bacteriophage**	**Percentage identity matrix (%)**				
F_podovirus	100	47	47	47	47
B_podovirus	47	100	99	99	99
**R_podovirus**	47	99	**100**	100	100
D_podovirus	47	99	100	100	100
C_podovirus	47	99	100	100	100

Figure S2: Multiple nucleotide sequence alignment and percentage identity matrix (%) of the gene encoding DNA-dependent RNA polymerase in *Podoviridae* phages F, B, R, D and C (Smith and Huggins, 1982).
