# Supplementary material for: Biology and Genomics of an Historic Therapeutic Escherichia coli Bacteriophage Collection
Source: Front Microbiol. 2017 Aug 30;8:1652. doi: 10.3389/fmicb.2017.01652 (PMC5582158; doi:10.3389/fmicb.2017.01652)
Supplement: Supplementary file 3 [file Image_3.PDF]

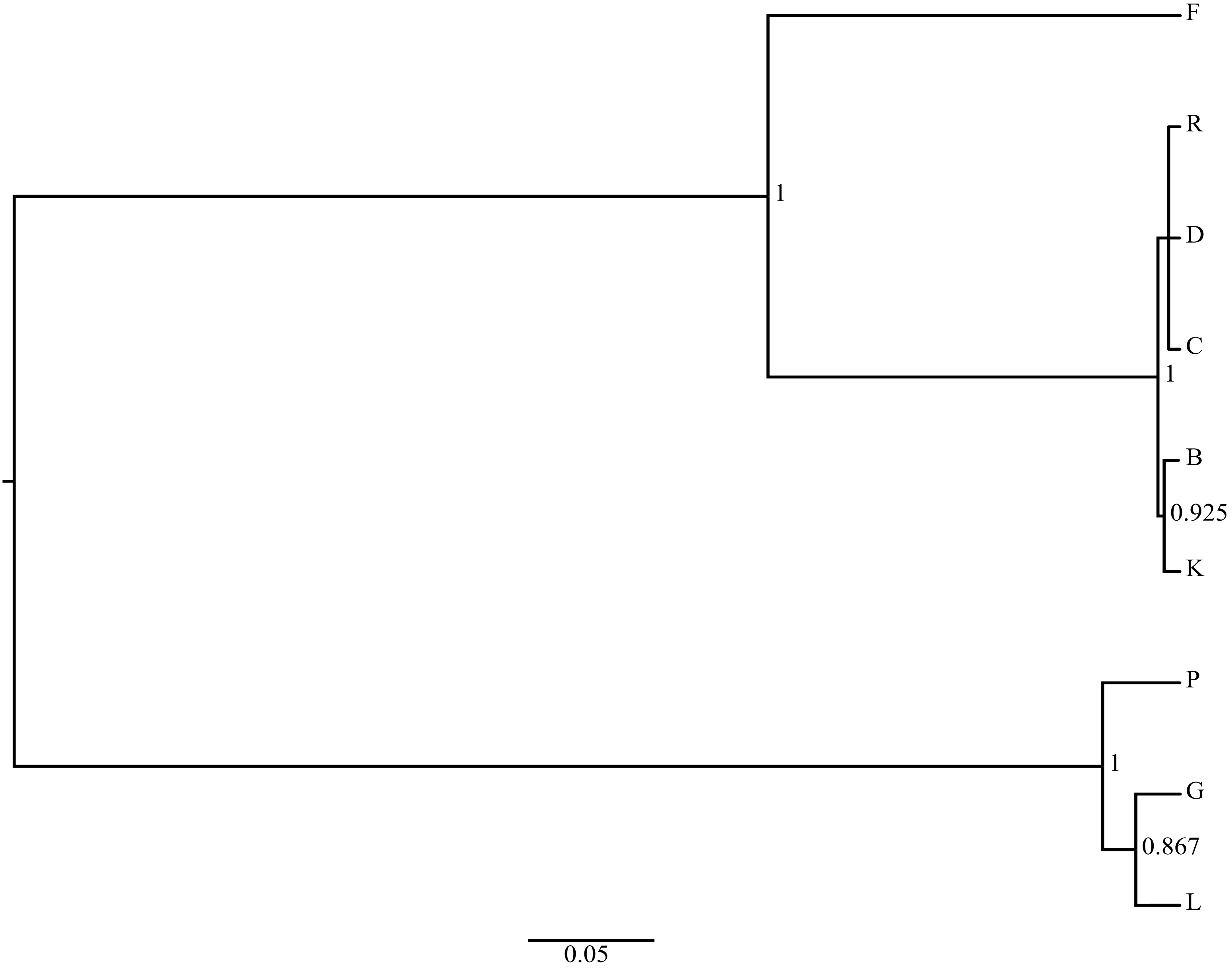

Figure S3: The maximum likelihood phylogenetic comparison of the gene encoding endosialidase in six *Podoviridae* and three *Siphoviridae* phages (Smith and Huggins, 1982).
