# Supplementary material for: Biology and Genomics of an Historic Therapeutic Escherichia coli Bacteriophage Collection
Source: Front Microbiol. 2017 Aug 30;8:1652. doi: 10.3389/fmicb.2017.01652 (PMC5582158; doi:10.3389/fmicb.2017.01652)
Supplement: Supplementary file 4 [file Image_4.PDF]

```
P_siphovirus    1 ------------------------------------------------------------
G_siphovirus    1 ------------------------------------------------------------
L_siphovirus    1 ------------------------------------------------------------
F_podovirus     1 ATGTCCACGATTACACAATTCCCTTCAGGAAACACTCAGTACAGGATTGAGTTCGACTAC
R_podovirus     1 ------------------------------------------------------------
C_podovirus     1 ------------------------------------------------------------
D_podovirus     1 ------------------------------------------------------------
B_podovirus     1 ------------------------------------------------------------
K_podovirus     1 ------------------------------------------------------------
consensus       1

P_siphovirus    1 ------------------------------------------------------------
G_siphovirus    1 ------------------------------------------------------------
L_siphovirus    1 ------------------------------------------------------------
F_podovirus    61 CTAGCCAGAACGTTTGTTGTTGTTACGCTGGTGAATAGCTCTAACCCTACCCTGAACCGT
R_podovirus     1 ------------------------------------------------------------
C_podovirus     1 ------------------------------------------------------------
D_podovirus     1 ------------------------------------------------------------
B_podovirus     1 ------------------------------------------------------------
K_podovirus     1 ------------------------------------------------------------
consensus      61

P_siphovirus    1 ------------------------------------------------------------
G_siphovirus    1 ------------------------------------------------------------
L_siphovirus    1 ------------------------------------------------------------
F_podovirus   121 GTACTGGAAGTTGGTCGAGATTACCGATTCCTTAATCCAACGATGATTGAGATGTTGGTT
R_podovirus     1 ------------------------------------------------------------
C_podovirus     1 ------------------------------------------------------------
D_podovirus     1 ------------------------------------------------------------
B_podovirus     1 ------------------------------------------------------------
K_podovirus     1 ------------------------------------------------------------
consensus     121

P_siphovirus    1 ----------------ATGTCAAGCGGATGCGGTGACG----------------TTTTAA
G_siphovirus    1 ----------------ATGTCAAGCGGATGCGGTGACG----------------TTTTAA
L_siphovirus    1 ----------------ATGTCAAGCGGATGCGGTGACG----------------TTTTAA
F_podovirus   181 GACCAATCAGGTTTCGACATCGTTCGTATTCACCGTCAGACTGGAACTGACTTAGTGGTA
R_podovirus     1 ------------------------------------------------------------
C_podovirus     1 ------------------------------------------------------------
D_podovirus     1 ------------------------------------------------------------
B_podovirus     1 ------------------------------------------------------------
K_podovirus     1 ------------------------------------------------------------
consensus     181

P_siphovirus   29 GCCTGGCGGATTTAC-------AAACCGCTAAGAAACACCAGATTTTCGAAGCCGAAGTT
G_siphovirus   29 GCCTGGCGGATTTAC-------AAACCGCCAAGAAACATCAGATTTTCGAGGCCGAGGTT
L_siphovirus   29 GCCTGGCGGATTTAC-------AAACCGCCAAGAAACATCAGATTTTCGAGGCCGAGGTT
F_podovirus   241 GACTTCAGGAATGGCTCAGTGTTGACAGCTAGTGACCTGACCAATTCAGAGCTTCAGGCT
R_podovirus     1 ------------------------------------------------------------
C_podovirus     1 ------------------------------------------------------------
D_podovirus     1 ------------------------------------------------------------
B_podovirus     1 ------------------------------------------------------------
K_podovirus     1 ------------------------------------------------------------
consensus     241

P_siphovirus   82 ATCA---------------------------CCGGTAAATCCGGTGGTG---TTGCTACT
G_siphovirus   82 ATCA---------------------------CTGGCAAATCCGGCGGTG---TCGCCGGT
L_siphovirus   82 ATCA---------------------------CTGGCAAATCCGGCGGTG---TCGCCGGT
F_podovirus   301 ATCCATATTGCAGAAGAAGGTCGAGACCAAACGGTTGACTTAGCGAAGGAATATGCCGAT
R_podovirus     1 ------------------------------------------------------------
C_podovirus     1 ------------------------------------------------------------
D_podovirus     1 ------------------------------------------------------------
B_podovirus     1 ------------------------------------------------------------
K_podovirus     1 ------------------------------------------------------------
```

```
consensus     301

P_siphovirus  112 GGTGCTGATATTGATTACGCAACAAATCAGGTTACAGGGCAGACGCAGAAGACACTCCCC
G_siphovirus  112 GGTGCCGATATTGATTACGCGACAAATCAGGTTACTGGACAGACCCAGAAGACGTTACCT
L_siphovirus  112 GGTGCCGATATTGATTACGCGACAAATCAGGTTACTGGACAGACCCAGAAGACGTTACCT
F_podovirus   361 GCTGCTGGTAGCTCTGCTGGCAACGCTAAGGATAGCGA--------GGACGAAGCACGCC
R_podovirus     1 ------------------------------------------------------------
C_podovirus     1 ------------------------------------------------------------
D_podovirus     1 ------------------------------------------------------------
B_podovirus     1 ------------------------------------------------------------
K_podovirus     1 ------------------------------------------------------------
consensus     361

P_siphovirus  172 GCCGTGTTGCGTGACGCAGGTTTTAAACTGGCAGATTTTGATTTCACGTCAGGCGGTACA
G_siphovirus  172 GCCGTGTTGCGTGATGCTGGTTTTAAACTGGCAGATTTTGATTTCACGTCAGGCGGTACA
L_siphovirus  172 GCCGTGTTGCGTGATGCTGGTTTTAAACTGGCAGATTTTGATTTCACGTCAGGCGGTACA
F_podovirus   413 GAATCGCTGCGAGTATCA---GGGCAGCTGGTCTAATTGGCTATATTACCCG----TCGC
R_podovirus     1 ------------------------------------------------------------
C_podovirus     1 ------------------------------------------------------------
D_podovirus     1 ------------------------------------------------------------
B_podovirus     1 ------------------------------------------------------------
K_podovirus     1 ------------------------------------------------------------
consensus     421

P_siphovirus  232 CTATCCGCTAACGACCGAAACACCGCAGTTT-TGTGGCCGTCGT--CCTCTGGCGGTGAC
G_siphovirus  232 CTATCCGCTAACGACCGAAACACCGCAGTTT-TGTGGCCGTCGT--CCTCTGGCGGTGAC
L_siphovirus  232 CTATCCGCTAACGACCGAAACACCGCAGTTT-TGTGGCCGTCGT--CCTCTGGCGGTGAC
F_podovirus   466 TCCTTCGAGAAAGGCTACAACGTTACAACATGGAGCGAGGTCCTGCTATGGGAAGAGGAT
R_podovirus     1 ------------------------------------------------------------
C_podovirus     1 ------------------------------------------------------------
D_podovirus     1 ------------------------------------------------------------
B_podovirus     1 ------------------------------------------------------------
K_podovirus     1 ------------------------------------------------------------
consensus     481

P_siphovirus  289 GGGGATTGGTACTATTGGTTGGGTGCTCTACCAAAAATTATCCCCGCGTTATCTTCTCCG
G_siphovirus  289 GGGGATTGGTACTATTGGTTGGGCGCTCTGCCAAAAACTATCCCGGCGTCATCTTCTCCG
L_siphovirus  289 GGGGATTGGTACTATTGGTTGGGTGCTCTACCAAAAATTATCCCAGCGTCATCTTCTCCG
F_podovirus   526 GGTGATTATTACCGCTGGGATGGTACGCTTCCAAAGAACGTTCCTGCTGGTTCAACTCCT
R_podovirus     1 ------------------------------------------------------------
C_podovirus     1 ------------------------------------------------------------
D_podovirus     1 ------------------------------------------------------------
B_podovirus     1 ------------------------------------------------------------
K_podovirus     1 ------------------------------------------------------------
consensus     541

P_siphovirus  349 CAGTCCACCGGTGGCGTGGCAGATGGGGCGTGGCAGCCCGTCGGGTCGGTCATACTGCGG
G_siphovirus  349 CAGTCTACAGGAGGCGTGTCCGAAGGGGCCTGGCAACGCGCCGGGTCGGTCGTACTGAGG
L_siphovirus  349 CAGTCCACCGGTGGCGTGGCAGATGGGGCGTGGCAGCCTGTCGGGTCGGTCATACTGCGG
F_podovirus   586 GAAACTTCCGGTGGGATTGGATTAGGTGCGTGGGTTAGTGTTGGTGATGCGGCTTTAAGA
R_podovirus     1 -----------------ATGATTCAAAGACTAGGT-----------------TCTTCATT
C_podovirus     1 -----------------ATGATTCAAAGACTAGGT-----------------TCTTCATT
D_podovirus     1 -----------------ATGATTCAAAGACTAGGT-----------------TCTTCATT
B_podovirus     1 -----------------ATGATTCAAAGACTAGGT-----------------TCTTCATT
K_podovirus     1 -----------------ATGATTCAAAGACTAGGT-----------------TCTTCATT
consensus     601                  ..........*..*.*..                 ...*....

P_siphovirus  409 CAGCAAATAT-CTGATGTAAACGGTGCAATTCTGTACCCAGATTTACATATGGCGCGATG
G_siphovirus  409 CAGCAAATAT-CTAATGCGAACGGCGCAATTCTGTACCCAGATTTACATGTGGCGCGATG
L_siphovirus  409 CAGCAAATAT-CTGATGTAAACGGTGCAATTCTGTACCCAGATTTACATGTGGCGCGATG
F_podovirus   646 AGTCAGATTTCAAAC-CCGGAAGGGGCAATACTCTACCCTGAATTACATAGAGCACGCTG
R_podovirus    27 AGTTAAATTCAAGAGTAAAATAGCAGGTGCAATCTGGCGTAACTTGGATGACAAGCTCAC
C_podovirus    27 AGTTAAATTCAAGAGTAAAATAGCAGGTGCAATCTGGCGTAACTTGGATGACAAGCTCAC
D_podovirus    27 AGTTAAATTCAAGAGTAAAATAGCAGGTGCAATCTGGCGTAACTTGGATGACAAGCTCAC
B_podovirus    27 AGTTAAATTCAAGAGTAAAATAGCAGGTGCAATCTGGCGTAACTTGGATGACAAGCTCAC
K_podovirus    27 AGTTAAATTCAAGAGTAAAATAGCAGGTGCAATCTGGCGTAACTTGGATGACAAGCTCAC
```

```
consensus     661 ....*.**..............*..*......*.*..*...*.**..**......*....

P_siphovirus  468 GAAGGACGAATACGACCCGCGCGCATGGGGTGCCGTGGGCGACGGTGTAACTGATGATAC
G_siphovirus  468 GAAGGACGAATACGACCCGCGCGCATGGGGCGCCGTGGGCGACGGCGTAACTGATGATAC
L_siphovirus  468 GAAGGACGAATACGACCCGCGCGCATGGGGCGCCGTGGGCGACGGCGTAACTGATGATAC
F_podovirus   705 GCTTGATGAAAAAGATGTTCGTGGTTGGGGTGCTAAAGGGGATGGTGTCACTGATGATAC
R_podovirus    87 CG---AGGTTGTATCGCTTAAAGATTTTGGAGCCAAAGGTGATGGTAAGACAAACGACCA
C_podovirus    87 CG---AGGTTGTATCGCTTAAAGATTTTGGAGCCAAAGGTGATGGTAAGACAAACGACCA
D_podovirus    87 CG---AGGTTGTATCGCTTAAAGATTTTGGAGCCAAAGGTGATGGTAAGACAAACGACCA
B_podovirus    87 CG---AGGTTGTATCGCTTAAAGATTTTGGAGCCAAAGGTGATGGTAAGACAAACGACCA
K_podovirus    87 CG---AGGTTGTATCGCTTAAAGATTTTGGAGCCAAAGGTGATGGTAAGACAAACGACCA
consensus     721 ..   *.*.............*..*..**.**....**.**.**....**..*.**...

P_siphovirus  528 TCAATCTATTTTTAATGTATTAGCAGCTTCTCCCAACAATTGGATTATAGACGGCAGGGG
G_siphovirus  528 CCAATCTATTTTTAATGTATTAGCCGCTTCACCCAACAATTGGATTATAGACGGCAGGGG
L_siphovirus  528 TCAATCTATTTTTAATGTATTAGCAGCTTCCCTCAACAATTGGATTATAGACGGCAGGGG
F_podovirus   765 AGCAGCGTTAACGAGCGCATTAAACGACACACCGGTTGGACAAAAGATTAATGGCAATGG
R_podovirus   144 AGATGCAGTAA---------ATGCAGCGATGGCTTCAGGTAAGAGAATTGACGGTGCTGG
C_podovirus   144 AGATGCAGTAA---------ATGCAGCGATGGCTTCAGGTAAGAGAATTGACGGTGCTGG
D_podovirus   144 AGATGCAGTAA---------ATGCAGCGATGGCTTCAGGTAAGAGAATTGACGGTGCTGG
B_podovirus   144 AGATGCAGTAA---------ATGCAGCGATGGCTTCAGGTAAGAGAATTGACGGTGCTGG
K_podovirus   144 AGATGCAGTAA---------ATGCAGCGATGGCTTCAGGTAAGAGAATTGACGGTGCTGG
consensus     781 .....*..*..         .....*.................*..**..*.**....**

P_siphovirus  588 TTTGACTTATAAAGTTTCCCAATTACCTGACATTAGTAAATTTAAGAATGCTGCATTCGT
G_siphovirus  588 CTTGACTTATAAAGTTTCCCAATTACCCGACATTAGTAAATTTAAGAATGCTGCATTCGT
L_siphovirus  588 TTTGACTTATAAAGTTTCCCAATTACCTGACATTAGTAAATTTAAGAATGCTGCATTCGT
F_podovirus   825 GAAGACATATAAGGTCACATCCCTGCCTGACATTAGCCGCTTCATCAACACTCGTTTCGT
R_podovirus   195 TGCTACTTACAAAGTATCATCTTTACCTGATATGGAGCGATTCTATAACACCCGCTTCGT
C_podovirus   195 TGCTACTTACAAAGTATCATCTTTACCTGATATGGAGCGATTCTATAACACCCGCTTCGT
D_podovirus   195 TGCTACTTACAAAGTATCATCTTTACCTGATATGGAGCGATTCTATAACACCCGCTTCGT
B_podovirus   195 TGCTACTTACAAAGTATCATCTTTACCTGATATGGAGCGATTCTATAACACCCGCTTCGT
K_podovirus   195 TGCTACTTACAAAGTATCATCTTTACCTGATATGGAGCGATTCTATAACACCCGCTTCGT
consensus     841 ....**.**.**.**..*.....*.**.**.**.......**....**..*....*****

P_siphovirus  648 TTACGAGCGCGTAGCCGGGCAACCGCTTACTTATGTAGCTGATGGCTTCTTTAACGGCTC
G_siphovirus  648 TTACGAGCGCGTAGCCGGGCAACCGCTTACTTATGTAGCTGATGGCTTCTTTAACAGCTC
L_siphovirus  648 TTACGAGCGCGTAGCCGGGCAACCGCTTACTTATGTAGCTGATGGCTTCTTTAACGGCTC
F_podovirus   885 TTATGAGCGCATTCCCGGACAACCTCTTTATTATGCCTCTGAAGAATTTGTTCAGGGTGA
R_podovirus   255 ATGGGAACGTTTAGCAGGTCAACCTCTTTACTATGTGAGTAAAGGTTTTATCAATGGTGA
C_podovirus   255 ATGGGAACGTTTAGCAGGTCAACCTCTTTACTATGTGAGTAAAGGTTTTATCAATGGTGA
D_podovirus   255 ATGGGAACGTTTAGCAGGTCAACCTCTTTACTATGTGAGTAAAGGTTTTATCAATGGTGA
B_podovirus   255 ATGGGAACGTTTAGCAGGTCAACCTCTTTACTATGTGAGTAAAGGTTTTATCAATGGTGA
K_podovirus   255 ATGGGAACGTTTAGCAGGTCAACCTCTTTACTATGTGAGTAAAGGTTTTATCAATGGTGA
consensus     901 .*..**.**..*..*.**.*****.***...****....*.*.*..**..*..*..*...

P_siphovirus  708 ACTAACTAAGGTGACGGATACCCCGTTCTACAACGCTTGGACGCAGGATAAAACTTTCGT
G_siphovirus  708 ACTAACTAAGGTGACGGATACCCCGTTCTACAACGCATGGACGCAGGATAAAACTTTCGT
L_siphovirus  708 ACTAACTAAGGTGACGGATACCCCGTTCTACAACGCATGGACGCAGGATAAAACTTTCGT
F_podovirus   945 GCTGTTCAAAATAACGGACACCCCTTATTATAATGCGTGGCCTCAGGATAAAGCGTTCGT
R_podovirus   315 ACTCTATAAAATCACGGATAACCCTTATTACAATGCTTGGCCTCAAGACAAAGCGTTTGT
C_podovirus   315 ACTCTATAAAATCACGGATAACCCTTATTACAATGCTTGGCCTCAAGACAAAGCGTTTGT
D_podovirus   315 ACTCTATAAAATCACGGATAACCCTTATTACAATGCTTGGCCTCAAGACAAAGCGTTTGT
B_podovirus   315 ACTCTATAAAATCACGGATAACCCTTATTACAATGCTTGGCCTCAAGACAAAGCGTTTGT
K_podovirus   315 ACTCTATAAAATCACGGATAACCCTTATTACAATGCTTGGCCTCAAGACAAAGCGTTTGT
consensus     961 .**....**..*.*****.*.***.*..**.**.**.***.*.**.**.***.*.**.**

P_siphovirus  768 CTACGACCATGTTATTTACGCCCCGTTTATGGCGGGCGAACGACATGGCGTTCAAAACTT
G_siphovirus  768 CTACGACAATGTTATTTACGCTCCGTTTATGGCAGGCGAACGACATGGCGTTCAAAACTT
L_siphovirus  768 CTACGACCATGTTATTTACGCCCCGTTTATGGCGGGCGAACGACATGGTGTTCAAAACTT
F_podovirus  1005 ATATGAGAATGTGATATATGCACCTTACATGGGTAGCGATCGTCATGGTGTTAGTCGTCT
R_podovirus   375 ATATGAGAACGTGATATATGCACCTTACATGGGTAGCGACCGTCATGGTGTTAGTCGTCT
C_podovirus   375 ATATGAGAACGTGATATATGCACCTTACATGGGTAGCGACCGTCATGGTGTTAGTCGTCT
D_podovirus   375 ATATGAGAACGTGATATATGCACCTTACATGGGTAGCGACCGTCATGGTGTTAGTCGTCT
B_podovirus   375 ATATGAGAACGTGATATATGCACCTTACATGGGTAGCGACCGTCATGGTGTTAGTCGTCT
K_podovirus   375 ATATGAGAACGTGATATATGCACCTTACATGGGTAGCGACCGTCATGGTGTTAGTCGTCT
```

```
consensus    1021 .**.**..*.**.**.**.**.**.*..****...****.**.*****.***.......*

P_siphovirus  828 ACATGTAGCTTGGGTTCGCTCTGGTGACGACGGGCAGACTTGGTCAATGCCTGAGTGGTT
G_siphovirus  828 ACATGTAGCTTGGGTTCGCTCTGGTGACGACGGGCAGACTTGGTCAATGCCTGAGTGGTT
L_siphovirus  828 ACATGTAGCTTGGGTTCGCTCTGGAGACGACGGGCAGACTTGGTCAATGCCTGAATGGTT
F_podovirus  1065 GCATGTATCATGGGTTAAGTCTGGTGATGATGGTCAAACATGGTCTACTCCAGAGTGGTT
R_podovirus   435 GCATGTATCATGGGTTAAGTCTGGTGACGATGGTCAAACATGGTCTACTCCAGAGTGGTT
C_podovirus   435 GCATGTATCATGGGTTAAGTCTGGTGACGATGGTCAAACATGGTCTACTCCAGAGTGGTT
D_podovirus   435 GCATGTATCATGGGTTAAGTCTGGTGACGATGGTCAAACATGGTCTACTCCAGAGTGGTT
B_podovirus   435 GCATGTATCATGGGTTAAGTCTGGTGACGATGGTCAAACATGGTCTACTCCAGAGTGGTT
K_podovirus   435 GCATGTATCATGGGTTAAGTCTGGTGACGATGGTCAAACATGGTCTACTCCAGAGTGGTT
consensus    1081 .******.*.******...*****.**.**.**.**.**.*****.*..**.**.*****

P_siphovirus  888 GACTCCAATCCATGCTGACTATAATGCTAGTGTTTCTGCGGACAGAGTTAACTACCACTG
G_siphovirus  888 GACCCCAATCCATGCTGACTATAATGCTAGTGTTTCCGCGGACAGAGTTAACTACCACTG
L_siphovirus  888 GACTCCAATCCATGCTGACTATAATGCTAGTGTTTCTACGGACAGAGTTAACTACCACTG
F_podovirus  1125 AACTGATCTGCATCCAGATTACCCTAC------------------AGTGAACTATCATTG
R_podovirus   495 AACTGATCTGCATCCAGATTACCCTAC------------------AGTGAACTATCATTG
C_podovirus   495 AACTGATCTGCATCCAGATTACCCTAC------------------AGTGAACTATCATTG
D_podovirus   495 AACTGATCTGCATCCAGATTACCCTAC------------------AGTGAACTATCATTG
B_podovirus   495 AACTGATCTGCATCCAGATTACCCTAC------------------AGTGAACTATCATTG
K_podovirus   495 AACTGATCTGCATCCAGATTACCCTAC------------------AGTGAACTATCATTG
consensus    1141 .**.....*.***.*.**.**...*.*                  ***.*****.**.**

P_siphovirus  948 CATGAGCATGGGTACGTGCGGCAACCGATTATATGCAGTTATAGAAACCCGCTACTTATC
G_siphovirus  948 CATGAGCATGGGTACGTGCGGCAACCGATTATATGCAGTTATAGAAACCCGCTACTTATC
L_siphovirus  948 CATGAGCATGGGTACGTGCGGCAACCGATTATATGCCGTTATAGAAACCCGCTACTTATC
F_podovirus  1167 TATGAGTATGGGTGTATGTCGCAACCGTCTGTTTGCCATGATTGAAACACGTACTTTAGC
R_podovirus   537 TATGAGTATGGGTGTATGTCGCAACCGTCTGTTTGCCATGATTGAAACACGTACTTTAGC
C_podovirus   537 TATGAGTATGGGTGTATGTCGCAACCGTCTGTTTGCCATGATTGAAACACGTACTTTAGC
D_podovirus   537 TATGAGTATGGGTGTATGTCGCAACCGTCTGTTTGCCATGATTGAAACACGTACTTTAGC
B_podovirus   537 TATGAGTATGGGTGTATGTCGCAACCGTCTGTTTGCCATGATTGAAACACGTACTTTAGC
K_podovirus   537 TATGAGTATGGGTGTATGTCGCAACCGTCTGTTTGCCATGATTGAAACACGTACTTTAGC
consensus    1201 .*****.******...**..*******..*.*.***..*.**.*****.**....***.*

P_siphovirus 1008 TAATATGCGGATGAAGAAGGCAGAACTTTGGTCACGCCCAATGCCGTATTTCAGACACCC
G_siphovirus 1008 TAATATGCGGATGAAGAAGGCAGAACTTTGGTCACGCCCAATGCCGTATTTTAGACACCC
L_siphovirus 1008 TAATATGCGGATGAAGAAGGCAGAACTTTGGTCACGCCCAATGCCGTATTTTAGACACCC
F_podovirus  1227 CAAGAACGCATTAACCAATTGTGCATTGTGGGATCGCCCCATGTCTCGTAGCCTGCATCT
R_podovirus   597 CAAGAACGCACTAACCAATTGTGCATTGTGGGATCGCCCTATGTCTCGTAGTCTGCATCT
C_podovirus   597 CAAGAACGCACTAACCAATTGTGCATTGTGGGATCGCCCTATGTCTCGTAGTCTGCATCT
D_podovirus   597 CAAGAACGCACTAACCAATTGTGCATTGTGGGATCGCCCTATGTCTCGTAGTCTGCATCT
B_podovirus   597 CAAGAACGCACTAACCAATTGTGCATTGTGGGATCGCCCTATGTCTCGTAGTCTGCATCT
K_podovirus   597 CAAGAACGCACTAACCAATTGTGCATTGTGGGATCGCCCTATGTCTCGTAGTCTGCATCT
consensus    1261 .**.*......*.*..**....*.*.*.***...*****.***.*...*......**.*.

P_siphovirus 1068 AACCGGCGGAATAACTATTAGTTCTGGCTCCACTACTGCTACCATTGTCATAGAAAATCA
G_siphovirus 1068 AACCGGCGGAATAACTATTAGTTCTGGCTCCACTACTGCTACCATTGTCATAGAAAATCA
L_siphovirus 1068 AACCGGCGGAATAACTATTAGTTCTGGCTCCACTACTGCTACCATTGTCATAGAAAATCA
F_podovirus  1287 TACTGGCGGTATCACTAAAGCTGCAAATCAGCGATATGCAACAATACATGTACCAGACCA
R_podovirus   657 TACTGGTGGTATCACTAAGGCTGCAAATCAGAGATATGCAACAATCCATGTACCTGATCA
C_podovirus   657 TACTGGTGGTATCACTAAGGCTGCAAATCAGAGATATGCAACAATCCATGTACCTGATCA
D_podovirus   657 TACTGGTGGTATCACTAAGGCTGCAAATCAGAGATATGCAACAATCCATGTACCTGATCA
B_podovirus   657 TACTGGTGGTATCACTAAGGCTGCAAATCAGAGATATGCAACAATCCATGTACCTGATCA
K_podovirus   657 TACTGGTGGTATCACTAAGGCTGCAAATCAGAGATATGCAACAATCCATGTACCTGATCA
consensus    1321 .**.**.**.**.****....*.*...........***.**.**.....**....*.**

P_siphovirus 1128 TGGCCTAAAAGCAGGAGATGCGGTTAACTTCTCTAATACATCGGCAACTGGCGTATCGGG
G_siphovirus 1128 TGGCCTAAAAGCAGGAGATGCGGTTAACTTCTCTAATACATCGGCAACTGGCGTATCGGG
L_siphovirus 1128 TGGCCTAAAAGCAGGAGATGCGGTTAACTTCTCTAATACATCGGCAACTGGCGTATCGGG
F_podovirus  1347 CGGACTATTCGTGGGTGATTTTGTTAACTTCTCTAATTCTGCGGTAACAGGTGTATCCGG
R_podovirus   717 CGGACTCTTCGTTGGTGATTTTGTTAACTTCTCTAACTCTGCGGTAACAGGTGTATCTGG
C_podovirus   717 CGGACTCTTCGTTGGTGATTTTGTTAACTTCTCTAACTCTGCGGTAACAGGTGTATCTGG
D_podovirus   717 CGGACTCTTCGTTGGTGATTTTGTTAACTTCTCTAACTCTGCGGTAACAGGTGTATCTGG
B_podovirus   717 CGGACTCTTCGTTGGTGATTTTGTTAACTTCTCTAACTCTGCGGTAACAGGTGTATCTGG
K_podovirus   717 CGGACTCTTCGTTGGTGATTTTGTTAACTTCTCTAACTCTGCGGTAACAGGTGTATCTGG
```

```
consensus    1381 .**.**....*..**.***...**************..*..***.***.**.*****.**

P_siphovirus 1188 TAATATGACGGTTTCGTCTATAATTAACCGAAATACGTTCACTGTTACGCTCTCAAGCCC
G_siphovirus 1188 TAATATGACGGTTTCGTCTATAATTAACCGAAATACGTTCACTGTTACGCTCTCAAGCCC
L_siphovirus 1188 TAATATGACGGTTTCGTCTATAATTAACCGAAATACGTTCACTGTTACGCTCTCAAGCCC
F_podovirus  1407 TGATATGACTGTTGCAACCGTAATAGATAAGGACAACTTCACGGTTCTTACACCCAACCA
R_podovirus   777 TGATATGAAGGTTGCAACAGTAATAGATAAGGACAACTTCACGGTTCTTACACCTAACCA
C_podovirus   777 TGATATGAAGGTTGCAACAGTAATAGATAAGGACAACTTCACGGTTCTTACACCTAACCA
D_podovirus   777 TGATATGAAGGTTGCAACAGTAATAGATAAGGACAACTTCACGGTTCTTACACCTAACCA
B_podovirus   777 TGATATGAAGGTTGCAACAGTAATAGATAAGGACAACTTCACGGTTCTTACACCTAACCA
K_podovirus   777 TGATATGAAGGTTGCAACAGTAATAGATAAGGACAACTTCACGGTTCTTACACCTAACCA
consensus    1441 *.******..***.*..*..****..*.....*.*..*****.***.......*.*.**.

P_siphovirus 1248 TGCGAGTTCCTCCATAAACAATGCGGGTGTTGCCTGGAACTTCGCAACGCGATTCTGGGA
G_siphovirus 1248 GGCGAGTTCCACCATAAACAATGCGGGTGTTACCTGGAACTTCGCAACGCGATTCTGGGA
L_siphovirus 1248 GGCGAGTTCCACCATAAACAATGCGGGTGTTACCTGGAACTTCGCAACGCGATTCTGGGA
F_podovirus  1467 GCAGACTTCAGATTTGAATAACGCTGGAACGAATTGGCACATGGGTACTTCTTTCCATAA
R_podovirus   837 GCAGACTTCAGATTTGAATAACGCTGGAAAGAGTTGGCACATGGGTACTTCTTTCCATAA
C_podovirus   837 GCAGACTTCAGATTTGAATAACGCTGGAAAGAGTTGGCACATGGGTACTTCTTTCCATAA
D_podovirus   837 GCAGACTTCAGATTTGAATAACGCTGGAAAGAGTTGGCACATGGGTACTTCTTTCCATAA
B_podovirus   837 GCAGACTTCAGATTTGAATAACGCTGGAAAGAGTTGGCACATGGGTACTTCTTTCCATAA
K_podovirus   837 GCAGACTTCAGATTTGAATAACGCTGGAAAGAGTTGGCACATGGGTACTTCTTTCCATAA
consensus    1501 ...**.***.....*.**.**.**.**.......***.**.*.*..**....***....*

P_siphovirus 1308 CAGCCCGTGGGAAATTACCGAATTGCCGGGGGTAGCATACTCCACTAACGCTGATTTGTG
G_siphovirus 1308 CAGCCCGTGGGAAATTACCGAATTGCCTGAGGTAGCATACTCCACTAACGCTGATTTGTG
L_siphovirus 1308 CAGCCCGTGGGAAATTACCGAATTGCCTGGGGTAGCATACTCCACTAACGCTGATTTGTG
F_podovirus  1527 GTCTCCATGGCGTAAGACGGATCTT----------------GGTC-----TAATCCCTAG
R_podovirus   897 ATCTCCTTGGCGTAAGACAGATCTT----------------GGTC-----TAATCCCTCG
C_podovirus   897 ATCTCCTTGGCGTAAGACAGATCTT----------------GGTC-----TAATCCCTCG
D_podovirus   897 ATCTCCTTGGCGTAAGACAGATCTT----------------GGTC-----TAATCCCTCG
B_podovirus   897 ATCTCCTTGGCGTAAGACAGATCTT----------------GGTC-----TAATCACTCG
K_podovirus   897 ATCTCCTTGGCGTAAGACAGATCTT----------------GGTC-----TAATCCCTCG
consensus    1561 ....**.***...*..**.**..*.                ...*     .........*

P_siphovirus 1368 TGTCACGGAGACACATAGTTTTGCGGTTATAGATGATACTAACTACACTATCGCTGTCGG
G_siphovirus 1368 TGTCACGGAGACACATAGTTTTGCGGTTATAGATGATGTTAACTACACTATCGCTGTCGG
L_siphovirus 1368 TGTCACGGAGACACATAGTTTTGCGGTTATAGATGATGTTAACTACACTATCGCTGTCGG
F_podovirus  1566 TGTCACAGAGGTGCACAGCTTTGCTACTATTGATAACA------ATGGCTTTGTTATGGG
R_podovirus   936 TGTCACAGAGGTGCATAGCTTTGCTACTATTGATAACA------ATGGCTTTGTTATGGG
C_podovirus   936 TGTCACAGAGGTGCATAGCTTTGCTACTATTGATAACA------ATGGCTTTGTTATGGG
D_podovirus   936 TGTCACAGAGGTGCATAGCTTTGCTACTATTGATAACA------ATGGCTTTGTTATGGG
B_podovirus   936 TGTCACAGAGGTGCATAGCTTTGCTACTATTGATAACA------ATGGCTTTGTTATGGG
K_podovirus   936 TGTCACAGAGGTGCATAGCTTTGCTACTATTGATAACA------ATGGCTTTGTTATGGG
consensus    1621 ******.***...**.**.*****...***.***.*..      *.....*.*.*.*.**

P_siphovirus 1428 CTACCACAACGGCGATGTGTCCCCTCGTCGTCTTGGCGTCTTGTATTTTAGTAACGTTTA
G_siphovirus 1428 CTATCACAACGGCGATGTGTCCCCTCGGCGTCTCGGTGTCTTGTATTTTAGTAACGTGTA
L_siphovirus 1428 CTATCACAACGGCGATGTGTCCCCTAGGCGTCTCGGTGTCTTGTATTTTAGTAACGTGTA
F_podovirus  1620 CTATCATCAAGGTGATGTAGCTCCACGAGAAGTTGGGCTTTTCTACTTCCCTGATGCTTT
R_podovirus   990 CTATCATCAAGGTGATGTAGCTCCACGAGAAGTTGGGCTTTTCTACTTCCCTGATGCTTT
C_podovirus   990 CTATCATCAAGGTGATGTAGCTCCACGAGAAGTTGGGCTTTTCTACTTCCCTGATGCTTT
D_podovirus   990 CTATCATCAAGGTGATGTAGCTCCACGAGAAGTTGGGCTTTTCTACTTCCCTGATGCTTT
B_podovirus   990 CTATCATCAAGGTGATGTAGCTCCACGAGAAGTTGGGCTTTTCTACTTCCCTGATGCTTT
K_podovirus   990 CTATCATCAAGGTGATGTAGCTCCACGAGAAGTTGGGCTTTTCTACTTCCCTGATGCTTT
consensus    1681 ***.**..*.**.*****..*.**..*.....*.**..*.**.**.**...*.*.*..*.

P_siphovirus 1488 TGACAACCCGGGCGTATTCGTTCGTCGCACTGTCGCTCAGGCATACGCTGATAACGCTTC
G_siphovirus 1488 TGACAATCCCGGCGTATTCGTTCGTCGCACTGTCGCCCAGGCATACGCTGATAACGCTTC
L_siphovirus 1488 TGACAATCCTGGCGTATTCGTTCGTCGCACTGTCGCTCAGGCATACGCTGACAACGCTTC
F_podovirus  1680 CAATAGCCCATCTAATTATGTTCGTCGTCGGATACCATCTGAGTATGAACCAGATGCGGC
R_podovirus  1050 CAATAGCCCATCTAATTATGTTCGTCGTCAGATACCATCTGAGTATGAACCAGATGCGGC
C_podovirus  1050 CAATAGCCCATCTAATTATGTTCGTCGTCAGATACCATCTGAGTATGAACCAGATGCGGC
D_podovirus  1050 CAATAGCCCATCTAATTATGTTCGTCGTCAGATACCATCTGAGTATGAACCAGATGCGGC
B_podovirus  1050 CAATAGCCCATCTAATTATGTTCGTCGTCAGATACCATCTGAGTATGAACCAGATGCGGC
K_podovirus  1050 CAATAGCCCATCTAATTATGTTCGTCGTCAGATACCATCTGAGTATGAACCAGATGCGGC
```

```
consensus     1741 ..*.*..**.......*..********.....*..*....*..**.*......*.**..*

P_siphovirus 1548 TGAACCGTGCGTGAAAAGTTATGGAGGTGTGCTCTACCTTACCACACGCGGCACATTACC
G_siphovirus 1548 TGAGCCGTGCGTGAAAAGTTATGGTGGTGTGTTATACCTTACCACACGCGGCACATTACC
L_siphovirus 1548 TGAGCCGTGCGTGAAAAGTTATGGTGGTGTGTTATACCTTACCACACGCGGCACATTACC
F_podovirus  1740 AGAGCCATGCATCAAGTACTATGACGGTGTATTATACCTTATCACTCGTGGCACTCGTGG
R_podovirus  1110 AGAGCCATGCATCAAGTACTATGACGGTGTATTGTACCTTATCACTCGTGGTACTCGTGG
C_podovirus  1110 AGAGCCATGCATCAAGTACTATGACGGTGTATTGTACCTTATCACTCGTGGTACTCGTGG
D_podovirus  1110 AGAGCCATGCATCAAGTACTATGACGGTGTATTGTACCTTATCACTCGTGGTACTCGTGG
B_podovirus  1110 AGAGCCATGCATCAAGTACTATGACGGTGTATTGTACCTTATCACTCGTGGTACTCGTGG
K_podovirus  1110 AGAGCCATGCATCAAGTACTATGACGGTGTATTGTACCTTATCACTCGTGGTACTTGTGG
consensus     1801 .**.**.***.*.**....****..*****..*.*******.***.**.**.**......

P_siphovirus 1608 TACTTCCCCTGGCTCTACACTGGCGATGAGCACCGATTCTGGGCAAAACTGGAGCTACTT
G_siphovirus 1608 TACTTCCCCTGGCTCTACACTGGCGATGAGCACCGATTCTGGGCAAAACTGGAGCTACTT
L_siphovirus 1608 TACTTCCCCTGGCTCTACACTGGCGATGAGCACCGATTCTGGGCAAAACTGGAGCTACTT
F_podovirus  1800 GGATAGACTAGGTAGCTCTTTGCACCGTAGTAGAGACATAGGTCAGACTTGGGAGTCACT
R_podovirus  1170 CGATCGACTAGGAAGCTCTCTGCATCGTAGTAGAGATATAGGTCAAACTTGGGAGTCACT
C_podovirus  1170 CGATCGACTAGGAAGCTCTCTGCATCGTAGTAGAGATATAGGTCAAACTTGGGAGTCACT
D_podovirus  1170 CGATCGACTAGGAAGCTCTCTGCATCGTAGTAGAGATATAGGTCAAACTTGGGAGTCACT
B_podovirus  1170 CGATCGACTAGGAAGCTCTCTGCATCGTAGTAGAGATATAGGTCAAACTTGGGAGTCACT
K_podovirus  1170 CGATCGACTAGGAAGCTCTCTGCATCGTAGTAGAGATATAGGTCAAACTTGGGAGTCACT
consensus     1861 ...*...*..**.....*..**......**.*..**....**.**.*..***...*...*

P_siphovirus 1668 GCGATTTCCGAACAATGTACATCACTCCAATCTTCCGTTCGCTAAAGTAGGTGACTATCT
G_siphovirus 1668 GCGATTTCCGAACAATGTACATCACTCCAATCTTCCGTTCGCTAAAGTAGGTGACTATCT
L_siphovirus 1668 GCGATTTCCGGACAATGTACATCACTCCAATCTTCCGTTCGCTAAAGTAGGTGACTATCT
F_podovirus  1860 GAGATTTCCACATAATGTTCATCATACTACATTACCTTTTGCTAAAGTAGGGGATGACCT
R_podovirus  1230 AAGATTTCCACATAATGTTCATCATACTACCTTACCTTTTGCTAAAGTAGGAGATGACCT
C_podovirus  1230 AAGATTTCCACATAATGTTCATCATACTACCTTACCTTTTGCTAAAGTAGGAGATGACCT
D_podovirus  1230 AAGATTTCCACATAATGTTCATCATACTACCTTACCTTTTGCTAAAGTAGGAGATGACCT
B_podovirus  1230 AAGATTTCCACATAATGTTCATCATACTACCTTACCTTTTGCTAAAGTAGGAGATGACCT
K_podovirus  1230 AAGATTTCCACATAATGTTCATCATACTACCTTACCTTTTGCTAAAGTAGGAGATGACCT
consensus     1921 ..*******..*.*****.*****..*.*...*.**.**.**********.**..*.**

P_siphovirus 1728 CTACATCTTTGGTACAGAACGTGCCTTTGGCGAGTGGGAAGGAGGCGAGCCGGACCGCCG
G_siphovirus 1728 CTACATCTTTGGTACAGAACGTGCCTTTGGTGAGTGGGAAGGTGGCGAGCAGGACAGCCG
L_siphovirus 1728 ATACATCTTTGGTACAGAACGTGCCTTTGGTGAGTGGGAAGGTGGCGAGCAGGACAGCCG
F_podovirus  1920 TATTATGTTTGGTTCAGAACGTGCAGAAAATGAATGGGAAGCAGGTGCACCAGATGATCG
R_podovirus  1290 TATTATGTTTGGTTCAGAACGTGCAGAAAATGAATGGGAAGCAGGTGCACCAGATGATCG
C_podovirus  1290 TATTATGTTTGGTTCAGAACGTGCAGAAAATGAATGGGAAGCAGGTGCACCAGATGATCG
D_podovirus  1290 TATTATGTTTGGTTCAGAACGTGCAGAAAATGAATGGGAAGCAGGTGCACCAGATGATCG
B_podovirus  1290 TATTATGTTTGGTTCAGAACGTGCAGAAAATGAATGGGAAGCAGGTGCACCCGATGATCG
K_podovirus  1290 TATTATGTTTGGTTCAGAACGTGCAGAAAATGAATGGGAAGCAGGTGCACCAGATGATCG
consensus     1981 ....**.******.**********......**.*******..**.*..*..**....**

P_siphovirus 1788 CTATGAAGGTTCGTATCCACGGACCTTTATGTGCAAAGTTAATGTGTCGTCCTGGCCTTC
G_siphovirus 1788 CTATGAAGGTTCGTATCCGCGGACCTTTATGTGCAAAGTTAATGTGTCGTCCTGGCCTTC
L_siphovirus 1788 CTATGAAGGTTCGTATCCGAGGACCTTTATGTGCAAAGTTAATGTGTCGTCCTGGCCTTC
F_podovirus  1980 TTACAAGGCATCTTATCCTCGCACCTTCTATGCACGCTTGAATGTA------AACAATTG
R_podovirus  1350 TTACAAGGCATCTTATCCTCGTACCTTCTATGCACGATTGAATGTA------AACAAGTG
C_podovirus  1350 TTACAAGGCATCTTATCCTCGTACCTTCTATGCACGATTGAATGTA------AACAAGTG
D_podovirus  1350 TTACAAGGCATCTTATCCTCGTACCTTCTATGCACGATTGAATGTA------AACAAGTG
B_podovirus  1350 TTACAAGGCATCTTATCCTCGTACCTTCTATGCACGATTGAATGTA------AACAAGTG
K_podovirus  1350 TTACAAGGCATCTTATCCTCGTACCTTCTATGCACGATTGAATGTA------AACAAGTG
consensus     2041 .**..*.*..**.*****..*.*****..........*.*****.      ......*.

P_siphovirus 1848 ATCTTTAGATGATGTTCAGTGGTTTAATATTACCGACCAAATATATCAAGGTCATATTGT
G_siphovirus 1848 ATCTTTAGATGATGTTCAGTGGTTTAATATTACCGACCAAATATACCAGGGTCATATCGT
L_siphovirus 1848 ATCTTTAGATGATGTTCAGTGGTTTAATATTACCGACCAAATATACCAGGGTCATATCGT
F_podovirus  2034 GAATGCAGATGATATTGAATGGGTTAACATCACTGACCAGATTTATCAGGGCGGAATAGT
R_podovirus  1404 GAATGCAGATGACATTGAATGGGTTAACATCACAGACCAAATCTATCAGGGTGACATTGT
C_podovirus  1404 GAATGCAGATGACATTGAATGGGTTAACATCACAGACCAAATCTATCAGGGTGACATTGT
D_podovirus  1404 GAATGCAGATGACATTGAATGGGTTAACATCACAGACCAAATCTATCAGGGTGACATTGT
B_podovirus  1404 GAATGCAGATGACATTGAATGGGTTAACATCACAGACCAAATCTATCAGGGTGACATTGT
K_podovirus  1404 GAATGCAGATGACATTGAATGGGTTAACATCACAGACCAAATCTATCAGGGTGACATTGT
```

```
consensus     2101 ...*..******..**.*.***.****.**.**.*****.**.**.**.**....**.**

P_siphovirus 1908 TAACTCTGCTGTCGGCGTCGGCTCTGTCTGTGTTAAAGATGGCTGGTTGTACTATATCTT
G_siphovirus 1908 TAACACTAATGTCGGCGTCGGCTCGGTCTGTGTTAAAGATGGCTGGTTGTACTATATCTT
L_siphovirus 1908 TAACACTGCTGTCGGCGTCGGCTCGGTCTGTGTTAAAGATGGCTGGTTGTACTATATCTT
F_podovirus  2094 TAACTCAGGTGTAGGTGTTGGTTCGGTAGTTGTGAAGGATAATTACATCTACTACATGTT
R_podovirus  1464 GAACTCTAGTGTAGGTGTAGGTTCGGTAGTAGTTAAAGACAGCTACATTTACTATATCTT
C_podovirus  1464 GAACTCTAGTGTAGGTGTAGGTTCGGTAGTAGTTAAAGACAGCTACATTTACTATATCTT
D_podovirus  1464 GAACTCTAGTGTAGGTGTAGGTTCGGTAGTAGTTAAAGACAGCTACATTTACTATATCTT
B_podovirus  1464 GAACTCTAGTGTAGGTGTAGGTTCGGTAGTAGTTAAAGACAGCTACATTTACTATATCTT
K_podovirus  1464 GAACTCTAGTGTAGGTGTAGGTTCGGTAGTAGTTAAAGACAGCTACATTTACTATATCTT
consensus     2161 .***.*...***.**.**.**.**.**....**.**.**....*...*.*****.**.**

P_siphovirus 1968 TGGTGGGGAAGACTTCTTCTCTCCCTGGAGCATAGGGGATAACAGTGCGAAGTTATGGTA
G_siphovirus 1968 TGGTGGGGAAGACTTCTTCTCTCCCTGGAGCATAGGGGATAACAGTGCGAAGTTATGGTA
L_siphovirus 1968 TGGTGGGGAAGACTTCTTCTCTCCCTGGAGCATAGGGGATAACAGTGCGAGGTTATGGTA
F_podovirus  2154 TGGTGGAGAGGACCATTTTAACCCATGGACATATGGAGATAACTCAGCGAAGGATCCATT
R_podovirus  1524 TGGTGGAGAAAACCATTTCAACCCAATGACTTATGGTGACAACAAAGATAAAGACCCATT
C_podovirus  1524 TGGTGGAGAAAACCATTTCAACCCAATGACTTATGGTGACAACAAAGATAAAGACCCATT
D_podovirus  1524 TGGTGGAGAAAACCATTTCAACCCAATGACTTATGGTGACAACAAAGATAAAGACCCATT
B_podovirus  1524 TGGTGGAGAAAACCATTTCAACCCAATGACTTATGGTGACAACAAAGATAAAGACCCATT
K_podovirus  1524 TGGTGGAGAAAACCATTTCAACCCAATGACTTATGGTGACAACAAAGATAAAGACCCATT
consensus     2221 ******.**..**...**....**...**.....**.**.***...*..*........*.

P_siphovirus 2028 CAAACATGATGGGCATCCGGCTGACTTATATAGTTACCGGATAAAGATTGAAGACCACCC
G_siphovirus 2028 CAAACATGATGGGCATCCGGCTGACTTATATAGCTACCGGATAAAGATTGAAGACCACCC
L_siphovirus 2028 CAAACATGATGGGCATCCGGCTGACTTATATAGCTACCGGATAAAGATTGAAGACCACCC
F_podovirus  2214 CAAAGCAGATGGTCATCCCTCAGATTTGTATTGCTACAAAATGAAGATTGGCCCAAGCAA
R_podovirus  1584 TAAAGGTCATGGACACCCCACTGATATATACTGCTATAAGATGCAGATTGCAAATGACAA
C_podovirus  1584 TAAAGGTCATGGACACCCCACTGATATATACTGCTATAAGATGCAGATTGCAAATGACAA
D_podovirus  1584 TAAAGGTCATGGACACCCCACTGATATATACTGCTATAAGATGCAGATTGCAAATGACAA
B_podovirus  1584 TAAAGGTCATGGACACCCCACTGATATATACTGCTATAAGATGCAGATTGCAAATGACAA
K_podovirus  1584 TAAAGGTCATGGACACCCCACTGATATATACTGCTATAAGATGCAGATTGCAAATGACAA
consensus     2281 .***....****.**.**..*.**..*.**..*.**....**..******.......*..

P_siphovirus 2088 GTATGTATCCCGTGATTTCAAATACGGAGCAACGCCTAACCGTACACTACCTGTCGTCAT
G_siphovirus 2088 GTACGTATCACGTGATTTCAAATACGGAGCAACTCCTAACCGTACAATACCTGTCGCCAT
L_siphovirus 2088 GTACGTATCACGTGATTTCAAATACGGAGCAACTCCTAACCGTACAATACCTGTCGCCAT
F_podovirus  2274 TCATGTTTCCCGTGATTTCAGGTATGGTGCTGTACCTAACAGAGCTGTTCCCGTGTTTTT
R_podovirus  1644 TCGTGTATCTCGTAAGTTTACATATGGTGCAACTCCGGGTCAGGCTATACCTACCTTCAT
C_podovirus  1644 TCGTGTATCTCGTAAGTTTACATATGGTGCAACTCCGGGTCAGGCTATACCTACCTTCAT
D_podovirus  1644 TCGTGTATCTCGTAAGTTTACATATGGTGCAACTCCGGGTCAGGCTATACCTACCTTCAT
B_podovirus  1644 TCGTGTATCTCGTAAGTTTACATATGGTGCAACTCCGGGTCAGGCTATACCTACCTTCAT
K_podovirus  1644 TCGTGTATCTCGTAAGTTTACATATGGTGCAACTCCGGGTCAGGCTATACCTACCTTCAT
consensus     2341 ....**.**.***.*.**.*..**.**.**....**........*..*.**........*

P_siphovirus 2148 GGGGGTAGATGGTGTACGTCATGTAGAAGCACCTTTAACGTTTGATAATGATGTAAGCGT
G_siphovirus 2148 GGGGGTANATGGNNTACGCCATNTANAACNCCTTTAA-----------------------
L_siphovirus 2148 GGGGGTAGATGGTGTACGCCATGTAGAAGCACCTTTAACGTTTGACAATGATGTAAGCGT
F_podovirus  2334 TGATACGAATGGGATTCGCACTGTTCCTGCGCCAATGGAATTTACAGGGGA---------
R_podovirus  1704 GGGTACTGATGGTATTCGTACTATTCCTGCTCCACTTCACTTATCAGGAGAGGTAGTTGC
C_podovirus  1704 GGGTACTGATGGTATTCGTACTATTCCTGCTCCACTTCACTTATCAGGAGAGGTAGTTGC
D_podovirus  1704 GGGTACTGATGGTATTCGTACTATTCCTGCTCCACTTCACTTATCAGGAGAGGTAGTTGC
B_podovirus  1704 GGGTACTGATGGTATTCGTACTATTCCTGCTCCACTTCACTTATCAGGAGAGGTAGTTGC
K_podovirus  1704 GGGTACTGATGGTATTCGTACTATTCCTGCTCCACTTCACTTATCAGGAGAGGTAGTTGC
consensus     2401 .*......****..*.**...*.*.......*............................

P_siphovirus 2208 GTACTCCCTGCATGTAACGGGTCTGGAATACGATGG------TACACAACAGTCTGCCGT
G_siphovirus      ------------------------------------------------------------
L_siphovirus 2208 GTACTCCCTGCATGTAACGGGCTGGAATACGATGG------TACACAACAGTCTGCCGT
F_podovirus  2385 ------TTTGGGTTTAGGTCATGTGACCATTAGAGCCAGCACCAGTAGTAACATTCGCTC
R_podovirus  1764 CGCAGATATGACTGTCGAACACCTTACCCTGAAGGCAAGTACAAGTGCTAATATTCGCTC
C_podovirus  1764 CGCAGATATGACTGTCGAACACCTTACCCTGAAGGCAAGTACAAGTGCTAATATTCGCTC
D_podovirus  1764 CGCAGATATGACTGTCGAACACCTTACCCTGAAGGCAAGTACAAGTGCTAATATTCGCTC
B_podovirus  1764 CGCAGATATGACTGTCGAACACCTTACCCTGAAGGCAAGTACAAGTGCTAATATTCGCTC
K_podovirus  1764 CGCAGATATGACTGTCGAACACCTTACCCTGAAGGCAAGTACAAGTGCTAATATTCGCTC
```

```
consensus    2461 ............................................................

P_siphovirus 2262 GCGTGCACGGTTTGATGGTGATTACGGGCTGATTTCAAAAACAGTCCCCACTAAAACCCC
G_siphovirus      ------------------------------------------------------------
L_siphovirus 2262 GCGCGCACGGTTTGATGGTGATTACGGGCTGATTTCAAAAACAGTCCCCACTAAAACCCC
F_podovirus  2439 TGAAGTTTTAATGGAAGGTGAATATGGGTTTATCGGGAAATCTATACCGACTGATAACCC
R_podovirus  1824 AGAGATGCTGATGGAAGGTGAATATGGGTTCATTGGTAAGACTATACCAACGGATAACCC
C_podovirus  1824 AGAGATGCTGATGGAAGGTGAATATGGGTTCATTGGTAAGACTATACCAACGGATAACCC
D_podovirus  1824 AGAGATGCTGATGGAAGGTGAATATGGGTTCATTGGTAAGACTATACCAACGGATAACCC
B_podovirus  1824 AGAGATGCTGATGGAAGGTGAATATGGGTTCATTGGTAAGACTATACCAACGGATAACCC
K_podovirus  1824 AGAGATGCTGATGGAAGGTGAATATGGGTTCATTGGTAAGACTATACCAACGGATAACCC
consensus    2521 ............................................................

P_siphovirus 2322 ATCGAGTCAAAGACTGATAGTTAGCGGTGGTGATGGTGCGTCCAGTTCCGATGGTGCGTT
G_siphovirus      ------------------------------------------------------------
L_siphovirus 2322 ATCGGGTCAAAGACTGATAGTTAGCGGTGGTGATGGTGCATCCAGTTCCGATGGTGCGTT
F_podovirus  2499 GGCAGGGCAGCGCATCATATTTTGCGGGGGTGAAGGCACCAGTTCAACGACTGGCGCACA
R_podovirus  1884 AACCGCACAACGTGTTATCATCTCTGGGGGAGAGGGTACAGCAGCAGATACCGGTGCGCA
C_podovirus  1884 AACCGCACAACGTGTTATCATCTCTGGGGGAGAGGGTACAGCAGCAGATACCGGTGCGCA
D_podovirus  1884 AACCGCACAACGTGTTATCATCTCTGGGGGAGAGGGTACAGCAGCAGATACCGGTGCGCA
B_podovirus  1884 AACTGCCCAACGTGTCATCATCTCTGGTGGAGAGGGTACAGCAGCAGATACCGGTGCGCA
K_podovirus  1884 AACTGCCCAACGTGTCATCATCTCTGGTGGAGGGGGTACAGCAGCAGATACCGGTGCGCA
consensus    2581 ... .. ........ ........... ................................

P_siphovirus 2382 GCTGCAACTGTACGGGTCGAACCATGCAACGCCTAACAGGGCTATTATGTATGCGTCAGG
G_siphovirus      ------------------------------------------------------------
L_siphovirus 2382 GCTGCAACTGTACGGGGCAAACCATGCAAAGCCTAACAGGGCTATTATGTATGCGTCAGG
F_podovirus  2559 GATTACGCTTTATGGTGCAAATAACACCGACTCAAGGCGAATAGTTTACAATGGTGCTGA
R_podovirus  1944 AATTACCTTACATGGAGCAGGTTCTAGTACTTCTAGACGCGCAGTATATAATGCCAATGA
C_podovirus  1944 AATTACCTTACATGGAGCAGGTTCTAGTACTTCTAGACGCGCAGTATATAATGCCAATGA
D_podovirus  1944 AATTACCTTACATGGAGCAGGTTCTAGTACTTCTAGACGCGCAGTATATAATGCCAATGA
B_podovirus  1944 AATTACCTTACATGGAGCAGGTTCTAGTACTTCTAGACGCGCAGTATATAATGCCAATGA
K_podovirus  1944 AATTACCTTACATGGAGCAGGTTCTAGTACTTCTAGACGCGCAGTATATAATGCCAATGA
consensus    2641 ............................................................

P_siphovirus 2442 AGGCTTCTACTCATTCAACAATTTCCTTCCTTACTTAGACTCGCAAGTGTCTTTAGGTTC
G_siphovirus      ------------------------------------------------------------
L_siphovirus 2442 AGGCTTCTACTCATACAACAATTTTCTTCCTTACTTAGACTCGAAAGTGTCTTTAGGTTC
F_podovirus  2619 GCATCTGTTCCAGACTGCTGACGTTAAGCCTTATAACGATAACGTCACTGCGCTTGGTGG
R_podovirus  2004 GCACTTGTTCAAAAGTGGGGCTATCATGCCATACAATGATAACGTGTATCCTGCTGGAGG
C_podovirus  2004 GCACTTGTTCAAAAGTGGGGCTATCATGCCATACAATGATAACGTGTATCCTGCTGGAGG
D_podovirus  2004 GCACTTGTTCAAAAGTGGGGCTATCATGCCATACAATGATAACGTGTATCCTGCTGGAGG
B_podovirus  2004 GCACTTGTTCCAAAGCGGGGCTATCATGCCATACAATGATAACGTGTATCCTGCTGGCGG
K_podovirus  2004 GCACTTGTTCCAAAGCGGGGCTATCATGCCATACAATGATAACGTGTATCCTGCTGGCGG
consensus    2701 .......... .... ......................................... ..

P_siphovirus 2502 GGCAGGTAACAGGTGGACTACGGTGTACGCGGCCACCGGTACGATAAACACGTCCGATGG
G_siphovirus      ------------------------------------------------------------
L_siphovirus 2502 GGCAGGTAATAGGTGGACTACGGTGTACGCGGCCACCGGTACGATAAACACGTCTGATGG
F_podovirus  2679 GCCTAGCAACCGTTTCACCACTGCATACCTCGGAAGTAACCCTATTGTTACTTCTAACGG
R_podovirus  2064 CCCTCGTAATAGATTCACTACTATCCATCTTACAAGTGACCCTATTATTACGTCTGATGC
C_podovirus  2064 CCCTCGTAATAGATTCACTACTATCCATCTTACAAGTGACCCTATTATTACGTCTGATGC
D_podovirus  2064 CCCTCGTAATAGATTCACTACTATCCATCTTACAAGTGACCCTATTATTACGTCTGATGC
B_podovirus  2064 CCCTCGTAATAGATTCACTACTATCCATCTTACAAGTGACCCTATTATTACGTCTGATGC
K_podovirus  2064 CCCTCGTAATAGATTCACTACTATCCATCTTACAAGTGACCCTATTATTACGTCTGATGC
consensus    2761 ............................................................

P_siphovirus 2562 TACGCTTAAGACGAACAAAGAGGAAATTGAGGGACAACTCCTTACCGCATGGGAAGATAT
G_siphovirus      ------------------------------------------------------------
L_siphovirus 2562 TACACTTAAGACGAACAAAGAGGAAATTGAGGGACAACTCCTTACCGCATGGGAGGATAT
F_podovirus  2739 GGAGAAGAAAACAGAGCCGGTAGTTTTTGATGATGCTTTCCTGGATGCCTGGGGTGACGT
R_podovirus  2124 CACTCATAAGTATGGATCACAAGGTATTGATGAGAGTGTACTAAAGGCATGGGGTAAGGT
C_podovirus  2124 CACTCATAAGTATGGATCACAAGGTATTGATGAGAGTGTACTAAAGGCATGGGGTAAGGT
D_podovirus  2124 CACTCATAAGTATGGATCACAAGGTATTGATGAGAGTGTACTAAAGGCATGGGGTAAGGT
B_podovirus  2124 CACTCATAAGTATGGATCACAAGGTATTGATGAGGGTGTACTAAAGGCATGGGGCAAGGT
K_podovirus  2124 CACTCATAAGTATGGATCACAAGGTATTGATGAGGGTGTACTAAAGGCATGGGGCAAGGT
```

```
consensus    2821 ....................................................... ....

P_siphovirus 2622 ACATGTAATATCTTTTAAGTGGCTTGAAAGCCTAGCAATCAAGGGCGACACGGCAAGGAT
G_siphovirus      ------------------------------------------------------------
L_siphovirus 2622 ACATGTAATATCTTTTAAGTGGCTTGAAAGCCTAGCAATCAAGGGTGACACTGCAAGGAT
F_podovirus  2799 TCGCTACATCATGTACCAGTGGTTAGATGCTGTTCAACTGAAAGGCAATGATGCTCGTAT
R_podovirus  2184 TAGCTTCAAGCAGTACAAGTTGATTGGTGAGATGTCTCGTGGTGT------GCACCACAC
C_podovirus  2184 TAGCTTCAAGCAGTACAAGTTGATTGGTGAGATGTCTCGTGGTGT------GCACCACAC
D_podovirus  2184 TAGCTTCAAGCAGTACAAGTTGATTGGTGAGATGTCTCGTGGTGT------GCACCACAC
B_podovirus  2184 TGCCTTCAAGCAGTACAAGTTGATTGGAGAGATGTCTAGTGGTGT------GCATCACAC
K_podovirus  2184 TGCCTTCAAGCAGTACAAGTTGATTGGAGAGATGTCTAGTGGTGT------GCATCACAC
consensus    2881 ........................... ......... .......       ... ....

P_siphovirus 2682 ACACTTCGGTGTGATAGCCCAAGATGTGCGAGACATCTTGGTCAAACATGGG-CTTATGG
G_siphovirus      ------------------------------------------------------------
L_siphovirus 2682 ACACTTCGGTGTGATAGCCCAAGATGTGCGAGACATCTTGGTCAACCATGGG-CTTATGG
F_podovirus  2859 TCACTTCGGGGTTATTGCACAGCAAATTCGTGATGTATTCATTGCAC-ATGGTCTGATGG
R_podovirus  2238 TCACTTTGGTGTATTGGCACAAGACATTGTAGCTGCTTTTGCATCCGAAGGGTTAGATGC
C_podovirus  2238 TCACTTTGGTGTATTGGCACAAGACATTGTAGCTGCTTTTGCATCCGAAGGGTTAGATGC
D_podovirus  2238 TCACTTTGGTGTATTGGCACAAGACATTGTAGCTGCTTTTGCATCCGAAGGGTTAGATGC
B_podovirus  2238 TCACTTTGGTGTATTGGCACAAGACATTGTAGCTGCTTTTGCATCCGAAGGGTTAGATGC
K_podovirus  2238 TCACTTTGGTGTATTGGCACAAGACATTGTAGCTGCTTTTGCATCCGAAGGGTTAGATGC
consensus    2941 ............................................................

P_siphovirus 2741 AGAAAGATTCTACCG--------------ATTGTAAATATGCTTTCTTGTGCTACGACT-
G_siphovirus      ------------------------------------------------------------
L_siphovirus 2741 AGAAAGATTCTACCG--------------AGTGTAAATATGCTTTCTTGTGCTACGACT-
F_podovirus  2918 ATGAAAACAGTACGA--------------ATTGTCGTTATGCTGTTCTGTGCTATGACAA
R_podovirus  2298 AATCGACTTCGGCATTGTCTCCTTCGAAGAAGGTCAGTTTGGTGTTAGATATAGTGAAAT
C_podovirus  2298 AATCGACTTCGGCATTGTCTCCTTCGAAGAAGGTCAGTTTGGTGTTAGATATAGTGAAAT
D_podovirus  2298 AATCGACTTCGGCATTGTCTCCTTCGAAGAAGGTCAGTTTGGTGTTAGATATAGTGAAAT
B_podovirus  2298 AATCGACTTCGGCATTGTCTCCTTCGAAGAAGGTCAGTTTGGTGTTAGATATAGTGAAAT
K_podovirus  2298 AATCGACTTCGGCATTGTCTCCTTCGAAGAAGGTCAGTTTGGTGTTAGATATAGTGAAAT
consensus    3001 ............................................................

P_siphovirus 2786 ----CAATT-------------------------------------------------GA
G_siphovirus      ------------------------------------------------------------
L_siphovirus 2786 ----CAATT-------------------------------------------------GA
F_podovirus  2964 GTATCAACGGATGACCGATACGGTATTTTCGCACAATGAAATCGTTGAACACACTGACGA
R_podovirus  2358 CTTAATCTTGGAGGCT------GCTTATACTC-----GTTATCGTTTA-GACAAGTTAGA
C_podovirus  2358 CTTAATCTTGGAGGCT------GCTTATACTC-----GTTATCGTTTA-GACAAGTTAGA
D_podovirus  2358 CTTAATCTTGGAGGCT------GCTTATACTC-----GTTATCGTTTA-GACAAGTTAGA
B_podovirus  2358 CTTAATCTTGGAGGCT------GCTTATACTC-----GTTATCGTTTA-GACAAGTTAGA
K_podovirus  2358 CTTAATCTTGGAGGCT------GCTTATACTC-----GTTATCGTTTA-GACAAGTTAGA
consensus    3061 ................      ..........     ........... ...........

P_siphovirus 2793 AGCCGCGTATGATATTGATGAAGAT------GGCCAGACTGTA---CAAGTATCCCCTCC
G_siphovirus      ------------------------------------------------------------
L_siphovirus 2793 AGCTGCGTATGATATTGATGAAGAT------GGCCAGACTGTA---CAAGTATCCCCTCC
F_podovirus  3024 GGAGGGTAATGTCACTACTACGGAAGAACCTGTATACACCGAGGTGGTTATTCACGAAGA
R_podovirus  2406 GGATATGTATGCCACTAATAAAATC---A--GT-TAA-----------------------
C_podovirus  2406 GGATATGTATGCCACTAATAAAATC---A--GT-TAA-----------------------
D_podovirus  2406 GGATATGTATGCCACTAATAAAATC---A--GT-TAA-----------------------
B_podovirus  2406 GGAGATGTATGCCACTAATAAAACC---A--GT-TAA-----------------------
K_podovirus  2406 GGAGATGTATGCCACTAATAAAACC---A--GT-TAA-----------------------
consensus    3121 ... ................... .   .  .. ...

P_siphovirus 2844 CGGCGGCAGATGGGGGGTTAGGGCGGACCAGATGTTCTTCATAGAGATTGCCTATCAGCG
G_siphovirus      ------------------------------------------------------------
L_siphovirus 2844 CGGCGGCAGATGGGGGGTTAGGGCGGACCAGATGTTCTTCATAGAGATTGCCTATCAGCG
F_podovirus  3084 GGGTGAGGAGTGGGGTGTTCGGCCTGACGGAATTTTCTTTGCTGAAGCCGCTTATCAGAG
R_podovirus       ------------------------------------------------------------
C_podovirus       ------------------------------------------------------------
D_podovirus       ------------------------------------------------------------
B_podovirus       ------------------------------------------------------------
K_podovirus       ------------------------------------------------------------
```

```
consensus    3181

P_siphovirus 2904 TAAGAAGATGCGCGAGTTCGATAAGAGGTTGGCAGCACTAGAAGATAAATAA
G_siphovirus      ----------------------------------------------------
L_siphovirus 2904 TAAGAAGATGCGCGAGTTCGAGAAGAGGCTGGCAGCACTAGAAGATAAATAA
F_podovirus  3144 AAGAAAACTGGAAAGAATCGAAGCCAGACTTTCTGCTCTTGAACAGAAGTAA
R_podovirus       ----------------------------------------------------
C_podovirus       ----------------------------------------------------
D_podovirus       ----------------------------------------------------
B_podovirus       ----------------------------------------------------
K_podovirus       ----------------------------------------------------
consensus    3241
```

**Bacteriophage**	**Percentage identity matrix (%)**								
P_siphovirus	100	95	97	53	52	52	52	52	52
G_siphovirus	95	100	97	55	55	55	55	55	55
L_siphovirus	97	97	100	53	52	52	52	52	52
F_podovirus	53	55	53	100	92	76	76	75	75
**R_podovirus**	52	55	52	76	**100**	100	100	99	99
C_podovirus	52	55	52	76	100	100	100	99	99
D_podovirus	52	55	52	76	100	100	100	99	99
B_podovirus	52	55	52	75	99	99	99	100	100
K_podovirus	52	55	52	75	99	99	99	100	100

Figure S4: Multiple nucleotide sequence alignment and percentage identity matrix (%) of the gene encoding endosialidase in six *Podoviridae* and three *Siphoviridae* phages (Smith and Huggins, 1982).
